# Oligomerisation and stereoselective polymerisation of alkenes and alkynes using pyridyl-based Al(iii) catalysts

**DOI:** 10.1039/d5sc07624b

**Published:** 2026-01-21

**Authors:** Dipanjana Choudhury, Richard Danylyuk, Alexandros Terzopoulos, Natalie S. Potter, Oren A. Scherman, Jonathan M. Goodman, Dominic S. Wright

**Affiliations:** a Yusuf Hamied Department of Chemistry, University of Cambridge Lensfield Rd. Cambridge CB2 1EW UK oas23@cam.ac.uk jmg11@cam.ac.UK dsw1000@cam.ac.uk

## Abstract

Dealkylation of the dimers [R_2_Al(2-py′)]_2_ (2-py′ = 6-substituted pyridyl, R = Me, ^i^Bu) with [Ph_3_C][B(C_6_F_5_)_4_] gives the putative cations [RAl(2-py′)_2_(µ-R)AlR]^+^ which can polymerise a range of alkenes with a high degree of stereoregularity (syndiotacticity in the case of polystyrene) and which cylotrimerise terminal alkynes to trisubstituted benzenes and a fulvene. This is the first report of stereoslective Al(iii) polymerisation and of the cyclotrimerisation of alkynes by static catalysis using a main group metal.

## Abstract 2

The putative Al(iii) cationic catalysts [RAl(2-py′)_2_(µ-R)AlR]^+^ (2-py′ = 6-substituted pyridyl, R = Me, ^i^Bu) can polymerise terminal alkenes with a high degree of stereocontrol and cyclotrimerise terminal alkynes.

## Graphical abstract

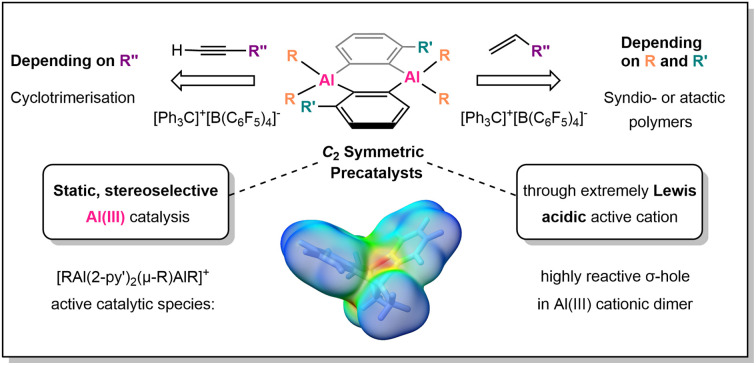

## Introduction

Sustainability is a central theme of concurrent chemistry, from alternative means of power generation and storage to chemical synthesis. In the area of catalysis, specifically, there is a major drive to move away from traditional, precious transition metal catalysts^1,2^ to more sustainable first-row transition metal and main group alternatives.^3–8^ Single-site aluminium catalysts have received significant attention within this context, as aluminium is the most abundant metal in the Earth's crust and offers associated cost advantages for industrial-scale applications over other main-group reagents.^9–11^ While research in this area is dominated by the search for redox catalysis using Al(i) (based on the Al(i)/Al(iii) couple),^12,13^ the more mature area of static catalysis based on Al(iii) (in which the metal oxidation state does not change) has already been successfully applied in a range of transition metal-mimetic reactions, including hydrogenation, dehydrocoupling, phosphination, hydrosilylation, hydroboration, as well as alkene, alkyne, diene and allylic ylide polymerisations.^9,10,14–16^

**Scheme 1:**
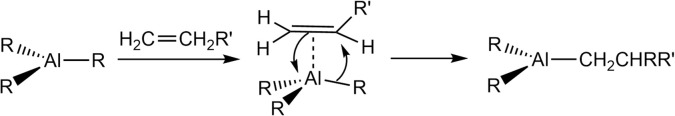
The fundamental steps involved in the carbalumination growth reaction. Further 1,2-insertion of alkenes (giving longer chains) is possible but not shown.

**Fig. 1:**
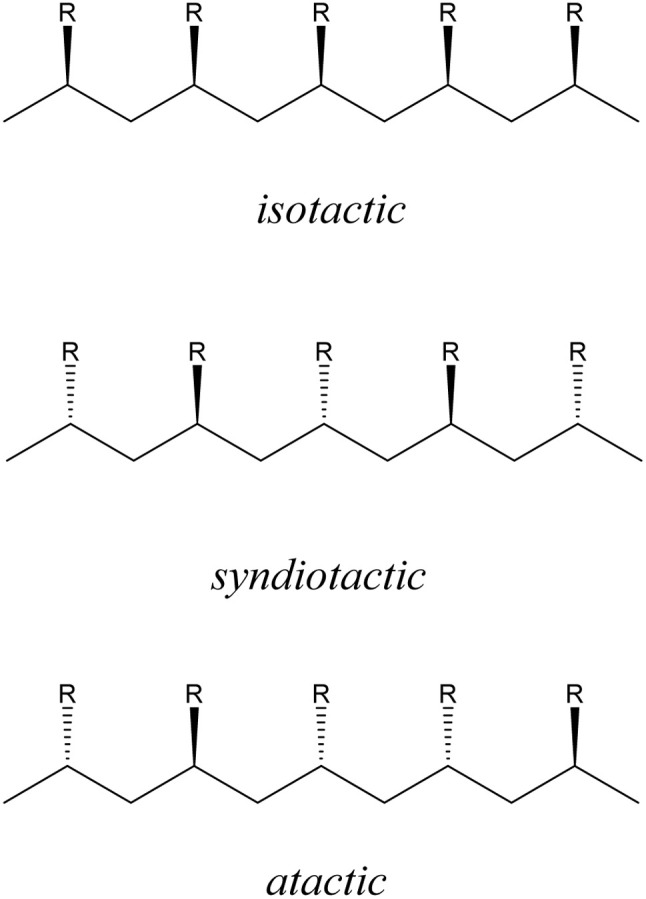
Polymerisation of α-olefins and their tacticity in polymers.

From its earliest beginnings with reactions such as the carbalumination growth reaction (Scheme 1),^13^ the high Lewis acidity of Al(iii) has made it especially useful in alkene and alkyne activation. In the absence of accessible d-orbitals for back-donation, alkene bonding to Al(iii) primarily involves π-donation to the vacant p-orbital of the metal, reducing the C=C bond strength and activating it to nucleophilic attack by another alkene molecule. A large range of catalytic homo- and co-polymerisations of olefins involving Al(iii) has been reported, and these have been the subject of several reviews.^10,17^ However, there are no examples involving control of the tacticity of the polymer backbone. In the absence of stereo-control, atactic polymers are produced, with a random arrangement of the R-groups, while stereocontrol can produce either isotactic (R-groups with the same orientation along the polymer chain) or syndiotactic (R-groups alternating along the polymer backbone) polymers (Fig. 1). Controlling tacticity is important as it directly affects the thermal and mechanical properties of polymeric materials. Conventionally, Lewis acidic Zr(iv) catalysts have been used to control tacticity of polyolefins, with the local ligand symmetry around the metal (in addition to the primary interaction with the growing polymer) dictating the face of the alkene incorporated (*si* and/or *re*) during chain propagation.^18,19^

Surprisingly few studies of main group metal systems have involved tacticity control in alkene polymerisation, despite corresponding recent progress on aluminium-mediated *cis*/*trans*-stereoregular diene and allylic ylide polymerisations by Hadjichristidis *et al.*^15,16^ For polyolefins, based on the conventional ligand design of common Zr(iv) catalysts, Harder *et al.* explored the effects of ligand symmetry and steric bulk on the tacticity of polystyrene using a series of homo- and heteroleptic benzyl/fluorenyl calcium catalysts, in the best case achieving around 95% syndiotactic polystyrene.^20–23^ However, in the absence of significant Lewis acidity of the metal and due to the high polarity of Ca–C bonds, a living anionic polymerisation mechanism operates. Clearly, the greater covalent nature of Al–C bonds together with the high Lewis acidity of Al(iii) should provide a much more rigid local ligand environment than s-block metals to support cationic stereoselective alkene insertion.

**Scheme 2:**
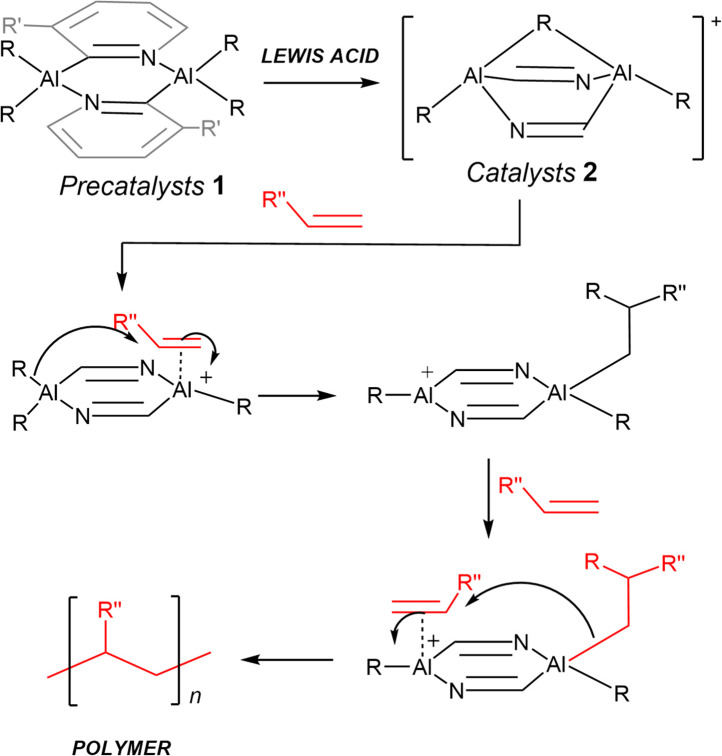
Proposed mechanism of polymerisation of terminal alkenes using the precatalyst 1. Note: the structure of the catalysts 2 is derived from DFT calculations reported by us earlier.^24^

We recently proposed that C_2_-symmetric *trans*-[RAl(2-py′)_2_(µ-R)AlR]^+^ cations (2) (2-py′ = 6-substituted pyridyl, R = Me, ^i^Bu), generated by the dealkylation of the dimers *trans*-[R_2_Al(2-py′)]_2_, (1) might be useful in stereocontrolled alkene polymerisation, *via* a co-operative mechanism involving both Al(iii) centres (Scheme 2).^24^ We show here that a high degree of syndiotacticity can indeed be obtained in the polymerisation of styrene using the readily prepared cations 2 (depending mainly on the Al-bonded R-group). These cations are also active in the catalytic cyclotrimerisation of terminal alkynes (phenyl and *t*-butyl acetylene) and can induce a high level of stereoregularity in the polymerisation of 1-hexene. To the best of our knowledge, this is the first report of stereocontrolled polymerisation of alkenes and the cyclotrimerisation of alkynes using an Al(iii) catalyst.

## Results and discussion

### Generation of catalysts 2

**Scheme 3:**
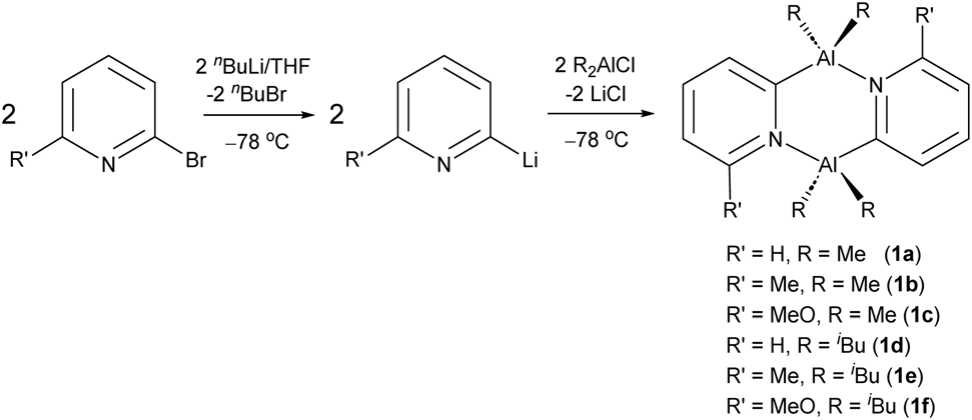
Synthesis of Al(iii) dimers of the type (2-py′ = 6-substituted pyridyl), the precatalysts used in polymerisation and cyclotrimerisation in the current study, including the compound numbering scheme.

In our previous report, we showed that Al(iii) dimers of the type [R_2_Al(2-py′)]_2_ (2-py′ = 6-substituted pyridyl, R = Me, ^i^Bu) are readily prepared by the one-step reaction of dialkylaluminium chlorides with 2-lithiopyridines (2-Li-py′) (Scheme 3).^24^ It was found that installing Me-groups at the 6-position of the bridging pyridyl rings increases the thermodynamic stability of the desired *trans* isomers (the precatalysts used in the current study) on steric grounds.

Our initial studies explored the demethylation of unsubstituted dimer [Me_2_Al(2-py)]_2_ (1a). NMR-scale reactions of 1 with B(C_6_F_5_)_3_ (BCF) in d_6_-benzene indicated incomplete reaction (^1^H NMR), with the formation of [(C_6_F_5_)_3_BMe]^−^ and [(C_6_F_5_)_2_BMe] (^11^B NMR). Although encouraging in that demethylation of 1a had clearly occurred, the formation of [(C_6_F_5_)_2_BMe] indicates that the [(C_6_F_5_)_3_BMe]^−^ anion is not stable in the presence of the cation. Similar observations had been made earlier by Smith *et al.* on demethylation of (TTP)-AlMe_2_ (TTP–H = 2-(*p*-tolylamino)-4-(*p*-tolylimino)-2-pentene) with BCF, with a C_6_F_5_ group from BCF being transferred to the Al(iii) producing (TTP)AlMe(C_6_F_5_).^25,26^ Consequently, we turned to the use of [Ph_3_C][B(C_6_F_5_)_4_] which has previously been employed in the dealkylation of a range of Al(iii) β-diketiminate complexes.^27^ As previously reported by us, ^1^H NMR studies of the dealkylation of 1a and 1c in d_6_-benzene or d_8_-toluene clearly showed the formation of Ph_3_CMe with no apparent decomposition of the [B(C_6_F_5_)_4_]^−^ anion.^24^ This finding is also supported by *in situ*^11^B and ^19^F NMR studies which only show the presence of the [B(C_6_F_5_)_4_]^−^ anion and no B(C_6_F_5_)_3_ decomposition product in any of the Lewis-acid-activated precatalyst solutions. In the case of the ^i^Bu derivatives, dealkylation results in the formation of Ph_3_CH and the evolution of isobutene (for ^1^H NMR spectra, see SI Fig. S3 and S4).^28^ This Lewis acid was therefore used in all of the subsequent polymerisation and cyclotrimerisation studies.

Before moving on to the discussion of studies on alkene and alkyne activation, it is important to note that we have been unable to recrystallise any of the cationic catalysts 2 using a range of conditions (varying solvents, temperatures, and adding strong donors to stabilise the cationic Al(iii) centres), with all attempts producing semisolids (even on removal of the reaction solvents under vacuum). As a result, the true nature of these species is not known at this stage. Our previously reported DFT calculations, however, suggest that dealkylation leads to alkyl-bridged arrangements [RAl(2-py′)_2_(µ-R)AlR]^+^ in model monomers (as depicted in Scheme 2).^24^ Importantly, the suggested mechanism of polymerisation involving co-operative transfer between the two bridged Al(iii) centres of the cations 2 (see Mechanism of Polymerisation, later) accounts for the high degree of stereoselectivity observed. This is in stark contrast to control experiments using [Ph_3_C][B(C_6_F_5_)_4_] in the absence of the precatalyst dimers (see SI experimental and Fig. S5) and supports the suggested nature of the active catalysts.

### Polymerisation of alkenes

#### Polystyrene

**Fig. 2:**
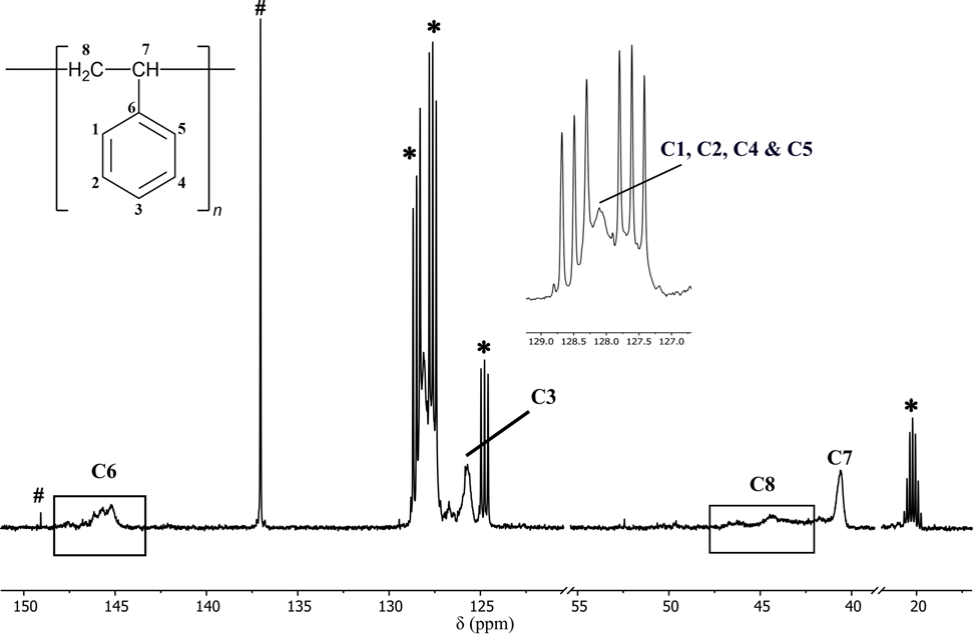
^13^C NMR (25 °C, d_8_-toluene, 126 MHz) spectrum of atactic polystyrene prepared using a 0.5 mol% precatalyst loading of 1a and [Ph_3_C][B(C_6_F_5_)_4_] at room temperature. The resonances are broad and overlapping and have been assigned based on existing literature.^21,30,31^ The signals marked as # can be best attributed to unreacted [Ph_3_C][B(C_6_F_5_)_4_], remnant toluene or potentially any unreacted styrene. *d_8_-toluene.

NMR-scale pilot studies were conducted using 5 and 10 mol% loadings of dimer [Me_2_Al(2-py)]_2_ (1a) activated by equimolar [Ph_3_C][B(C_6_F_5_)_4_] in d_8_-toluene under N_2_ (giving putative cation 2a). Exothermic reactions were observed on addition of neat styrene at room temperature and significantly more viscous solutions were formed, indicating the potential presence of polymeric products. ^1^H and ^13^C NMR spectroscopy showed broad resonances that are typical for atactic polystyrene in both cases (together with unreacted 1a). Control reactions under the same conditions using styrene only or 1a with no initiator did not result in polymerisation. Owing to the exothermic nature of the initial reaction and to encourage stereocontrol,^29^ the temperature was reduced to −15 °C using 0.5, 2.5, 5.0 and 10 mol% loadings of precatalyst 1a and a reaction time of 1 h. Quenching with methanol and removal of the volatiles under vacuum gave viscous semisolids which were shown to be atactic polystyrene by ^1^H and ^13^C NMR spectroscopy (Fig. 2).

**Fig. 3:**
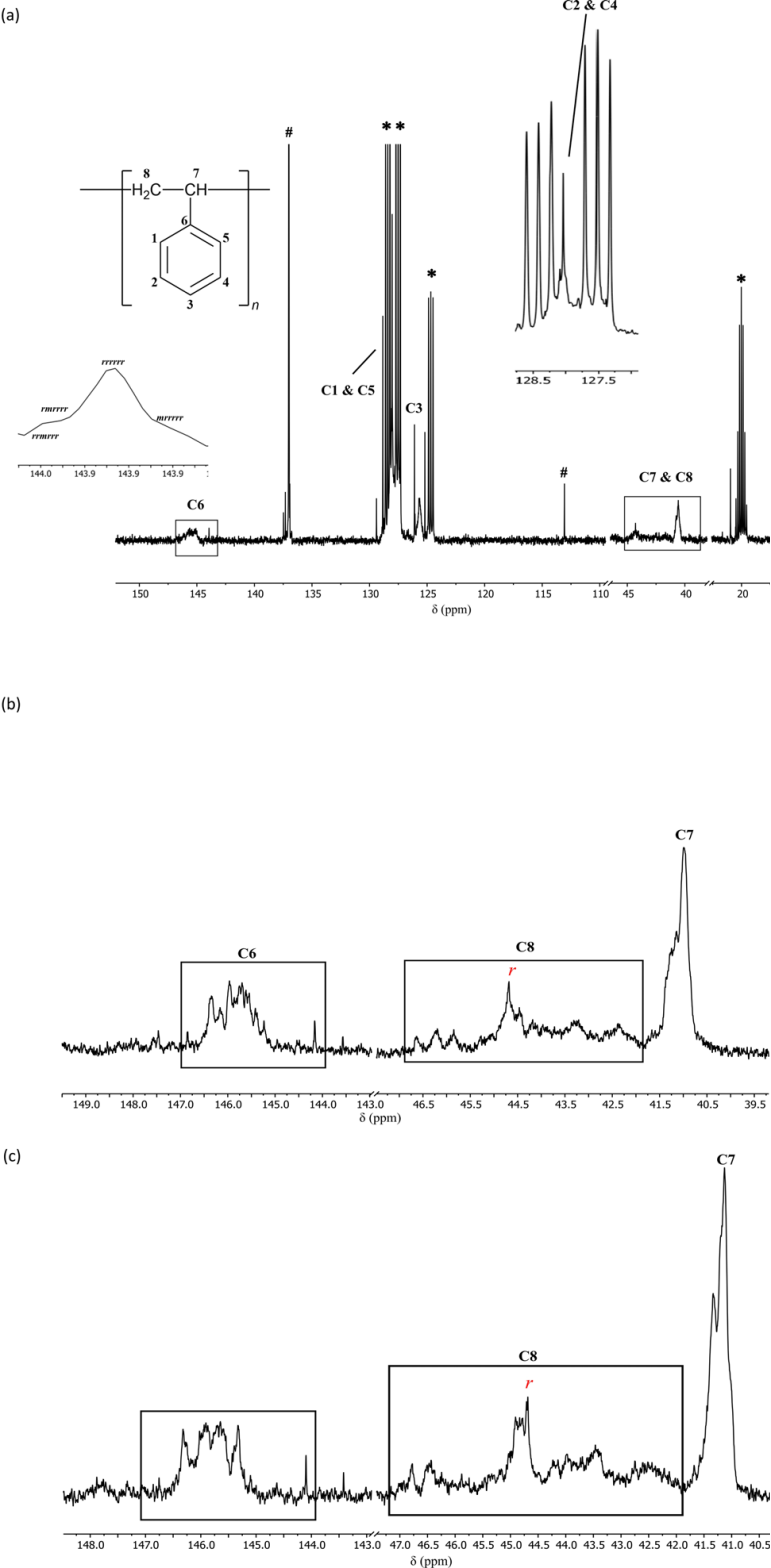
(a) ^13^C NMR (25 °C, d_8_-toluene, 126 MHz) spectrum of atactic + syndiotactic polystyrene prepared using 0.5 mol% catalytic loading of precatalyst 1e and [Ph_3_C][B(C_6_F_5_)_4_] at −15 °C depicting the signals for the aromatic ipso carbon and the methylene carbon. The broad signals for C6 and C8 represent the atactic component, while the sharper peaks represent the syndiotactic component. The sharp signal labelled with a red *r* marks the signal depicting a 100% probability of having *r* diads.^21,32^ The signals marked as # can be best attributed to unreacted [Ph_3_C][B(C_6_F_5_)_4_], remnant toluene or potentially any unreacted styrene. *d_8_-toluene. (b) ^13^C NMR (100 °C, d_2_-tetrachloroethane, 126 MHz, 1000 scans) showing the signals for the ipso carbon C6 and the methylene carbon C8. The sharp signal labelled with a red *r* marks the signal depicting a 100% probability of having *r* diads.^21,32^ (c) ^13^C NMR (100 °C, d_4_-*o*-dichlorobenzene, 126 MHz, 1000 scans) showing the signals for the ipso carbon C6 and the methylene carbon C8. The sharp signal labelled with a red *r* marks the signal depicting a 100% probability of having *r* diads.^21,32^

**Fig. 4:**
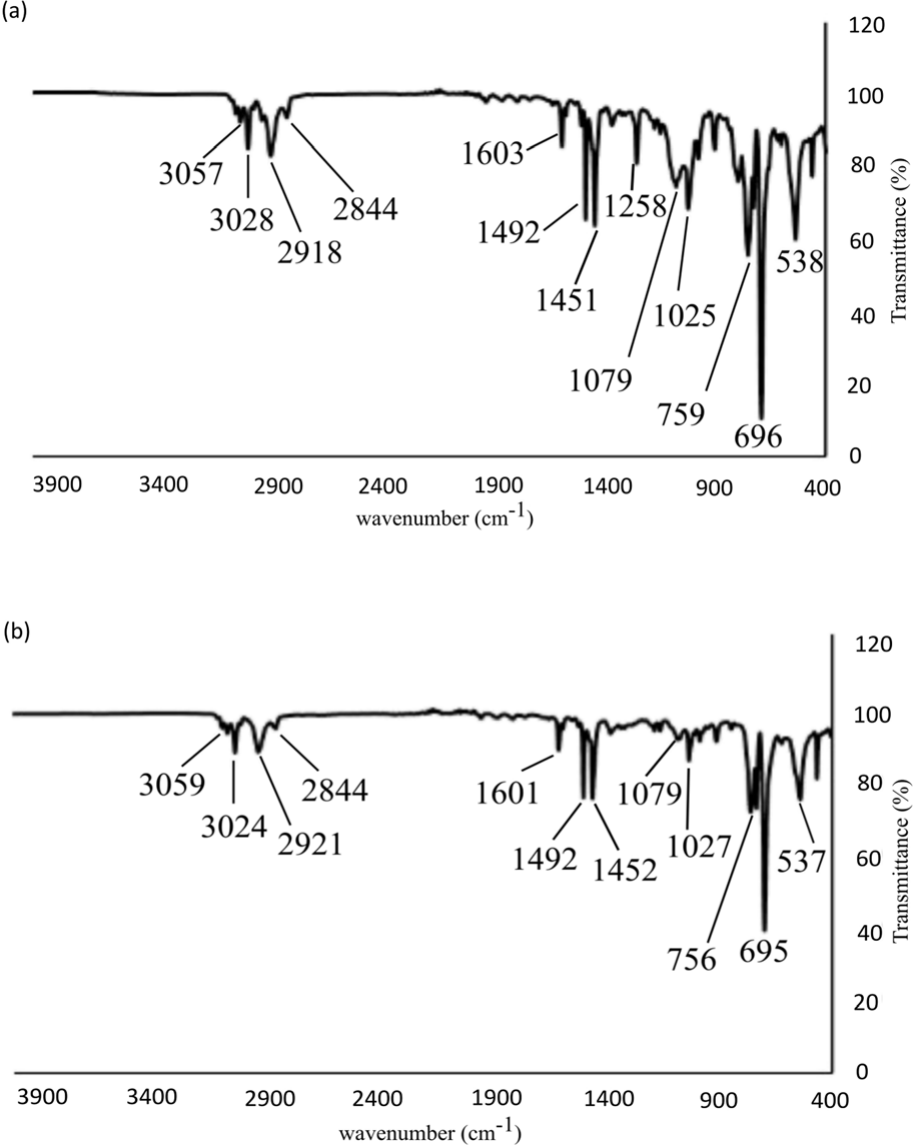
(a) IR spectrum of syndiotactic polystyrene prepared using a 0.5 mol% precatalyst loading of [^i^Bu_2_Al(6-Me-py)]_2_ (1e) and [Ph_3_C][B(C_6_F_5_)_4_] to generate cation 2e*in situ* at −15 °C. (b) IR spectra of atactic polystyrene prepared using a 0.5 mol% precatalyst loading of [Me_2_Al(6-MeO-py)]_2_ (1c) and [Ph_3_C][B(C_6_F_5_)_4_] to generate cation 2c*in situ* at 0 °C.

Reasoning that the Al-bonded Me-groups of 1a may not provide sufficient steric influence over the cationic Al(iii) reaction site, we turned to the more sterically hindered dimer [^i^Bu_2_Al(6-Me-2py)]_2_ (1e). In 1e, the steric effects at the Al(iii) and pyridyl rings are increased ([^i^Bu_2_Al(2-py)]_2_ (1d) was not used because it is a semi-solid at room temperature). The reactions at −15 °C for 48 h using 0.5–10 mol% loadings of 1e and the initiator gave similar stretchable materials after quenching, removal of volatiles and washing with methanol. Very encouragingly, although the polystyrene produced in all of these reactions contain atactic components, they also contain syndiotactic components, as confirmed by ^13^C NMR (Fig. 3) and IR spectroscopy (Fig. 4). The ratio of atactic to syndiotactic components could not be determined accurately because of the overlapping of the characteristic resonances in the ^1^H NMR spectrum. However, analysis of the ^13^C NMR spectrum allowed a clearer indication of the tacticity.

While the specific ^13^C NMR assignments for syndiotactic polystyrene have been debated, there is a wealth of literature on tacticity-based assignments.^31,33–39^ Here the initial tacticity-based assignments stated by Ishihara *et al.* were followed.^30^ For further confirmation, ^13^C NMR spectroscopy was conducted under the exact conditions outlined by Harder *et al.* (1000 scans at 100 °C in d_2_-tetrachloroethane and d_4_-*o*-dichlorobenzene; Fig. 3b and c, respectively).^21^ The presence of sharp peaks representing the aromatic ipso C (C6) in the *δ* 143.9–146 ppm range indicate the presence of a majority of *r* diads for up to heptad level assignments.^21,38^ For the methylene C (C8), sharp peaks at *δ* 44.29 ppm (Fig. 3a), 44.67 ppm (Fig. 3b) and 44.68 ppm (Fig. 3c) represent a polymeric chain with all *r* diads (from tetrad to hexad level).^21,38^ It can therefore be concluded that the use of 1e introduces a high level of regioregularity to the polymer chain, specifically one with a majority of *r* diads, thereby creating a large syndiotactic component (for further detailed discussion of assignments, see SI). Owing to the significantly lower molecular weight of these polymeric products and the low resolution of the NMR spectrum, assignments at levels higher than a diad were not attempted. Interestingly, for the 2.5 mol% loading of 1e, the ^13^C NMR spectrum of the polymeric product indicated an additional isotactic component, which appears further upfield of the syndiotactic and atactic resonances (ESI).^40^

Using [Me_2_Al(6-Me-2-py)]_2_ (1b), [Me_2_Al(6-MeO-2-py)]_2_ (1c) and [^i^Bu_2_Al(6-MeO-2-py)]_2_ (1f) as precatalysts (0 °C, 1 h) gave only atactic polystyrene for 1b and 1c, while 1f gave a mixture of atactic and syndiotactic (sharp ^13^C NMR peaks denoting syndiotactic linkages). The presence of the syndiotactic component in 1f was also confirmed by IR spectroscopy (ESI).^22,32,33^ The slightly higher temperature (compared to the aforementioned −15 °C) was used to encourage a faster reaction and potentially higher molecular weight products.

Fig. 4 compares the IR spectra of the polymeric products obtained using 1c (atactic) and 1e (syndiotactic). While the two polymers exhibit similar bands in their IR spectra for aliphatic and aromatic C–H and C=C stretching, and C–H bending vibrations, indicating a common polymeric backbone, most significantly, the weak band at 1258 cm^−1^ present in the polymer obtained from 1e is absent in that obtained from 1c. Ishihara *et al.* have reported that a peak at around 1220 cm^−1^ (close to that observed here) is diagnostic of syndiotactic polystyrene.^30^ The relative sharpness of the band at 538 cm^−1^ for the polystyrene obtained using 1e compared to that from 1c (537 cm^−1^) also confirms syndiotacticity as this peak is associated with the local *trans*–*trans* conformations. This conclusion is also supported by the observation of a sharper, more intense band at 1079 cm^−1^ for polystyrene obtained from 1e. The absence of the signature peaks for isotactic polystyrene (at 1364, 1314, 1297, and 1185 cm^−1^) discounts the presence of an isotactic component in either of the polymers obtained from 1c and 1e.^41^

Differential scanning calorimetry (DSC) measurements were performed to analyse the thermal behaviour of the polystyrene samples. The DSC traces revealed a glass transition temperature (*T*_g_) of 95.34 °C for the atactic polymeric product obtained from 1b and 105.12 °C for the syndiotactic product obtained using 1f (SI Fig. S23 and S24). These values agree with those reported for atactic and syndiotactic polystyrene, respectively, in the literature, complementing the NMR and IR characterisation.^42,43^

The results of the studies of 1a–c, 1e and 1f indicate that steric effects arising from the Al-bonded R-groups have the most significant result on stereoselectivity (rather than the R′-groups on the pyridyl rings). Turnover numbers (TONs) in the range 82–164 were determined for various precatalysts at different temperatures and loadings, illustrating that these reactions are indeed catalytic. These low values, however, suggest that the catalytic species themselves have a relatively short lifetime.

Table 1 shows the results of Gel Permeation Chromatography (GPC) studies of polystyrene formed using the range of precatalysts explored (see SI Fig. S8–S18 for GPC traces).

**Table 1:** GPC studies of selected polystyrene products using different dimeric precatalysts, activated using [Ph_3_C][B(C_6_F_5_)_4_]^a^

Precatalysts 1	Loading (mol%)	Temp. (^o^C)	Time (h)	*M*_n_ (g mol^−1^)	*M*_w_ (g mol^−1^)	*Đ*
1a	0.5	RT	1	2191	6514	2.9
	2.5	RT	1	1689	3595	2.1
	0.5	−15	1	5103	8687	1.7
	2.5	−15	1	5142	6030	1.2
1b	0.5	0	1	2118	10 740	5.0
1c	0.5	0	1	3515	13 602	3.9
1e	0.5	−15	48	5123	10 376	2.0
	2.5	−15	48	1411	2959	2.1
	5.0	−15	48	2045	5307	2.6
	10.0	−15	1	2613	3195	1.2
1f	0.5	0	1	1961	5929	3.0

a *M*_n_ = number average molecular weight, *M*_w_ = weight average molecular weight, *Đ* = dispersity determined by GPC (DMF, 0.1% LiBr, 40 °C) relative to polystyrene standards.

All the catalysts (1a–c, 1e and 1f) produce low molecular weight polystyrene (*M*_n_ < 10 000 g mol^−1^), although it should be noted that the reaction conditions in Table 1 are not optimised. These molecular weights are significantly lower than obtained using either transition metal or calcium-based systems (*ca. M*_n_ = 10^5^ g mol^−1^).^29^ Nonetheless, low molecular weight polystyrene has a number of important applications.^40,44,45^ Despite the limited data available so far, some overall conclusions can be made. Perhaps most significantly, it is clear from the data for 1a and 1e at −15 °C that the characteristics of the polymers produced (*i.e.*, *M*_n_, *M*_w_, and *Đ*) depend on the specific catalyst employed, illustrating that they do not involve a common catalytic species. The trend in the data for 1a and 1e shows that highest molecular weights are generally achieved at lower catalyst loadings and lower temperatures. This is as expected since a smaller number of polymers are generated initially, in the presence of a large excess of monomers, along with greater control of the polymerisation process at lower temperature.

#### Scope

Polymerisation studies were extended to 1-hexene and cyclohexene. For this purpose, we used precatalysts [Me_2_Al(2-py)]_2_ (1a) and [^i^Bu_2_Al(6-MeO-2-py)]_2_ (1f) to explore the extremes of the steric effects in the catalysts generated using [Ph_3_C][B(C_6_F_5_)_4_] as the initiator. In both cases, 10 mol% loadings of 1a and 1f were used, with toluene as the solvent. For 1-hexene, reactions were undertaken at room temperature for 30 min, while for cyclohexene more forcing conditions (80 °C for 24 h) were used. The latter reaction conditions were based on previous studies of the polymerisation of cyclohexene by Farona and Tsonis.^46^ As before, the polymeric products were isolated by quenching with methanol and washing the products with methanol before analysis.

**Fig. 5:**
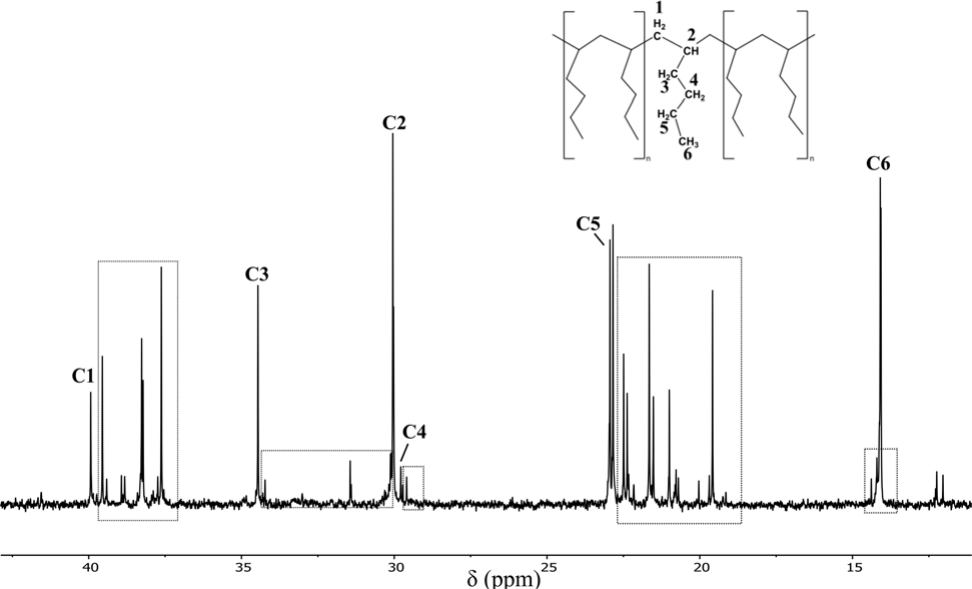
^13^C NMR (25 °C, d-chloroform, 126 MHz) spectrum of poly(1-hexene) prepared using a 10 mol% catalytic loading of 1f at room temperature. # vacuum grease.

The ^1^H NMR spectra of the poly(1-hexene) products obtained using 1a and 1f reveal a level of stereoregularity mixed with atactic components (SI), which was supported by ^13^C NMR spectroscopy.^47,48^ The six expected resonances for the monomer fragment are observed in the ^13^C NMR spectrum of poly(1-hexene) produced using 1f, in addition to several other broad peaks. The sharpness of the six primary peaks indicates a level of stereoregularity and, based on the values reported in the literature, the peak at *δ* 34.46 ppm can be attributed to the *rr* triad, thereby indicating that the polymer is primarily syndiotactic.^49^ As was seen in the above studies of polystyrene, the smaller resonances adjacent to the primary peaks can be attributed to monomer misinsertions (highlighted in Fig. 5). These minor resonances suggest the incorporation of some carbon chains with different methylene-backbone and side-chain lengths (unlike the regular C_4_-chains expected for ‘standard’ poly(1-hexene) chain synthesised using the Ziegler–Natta process).

The ^1^H NMR spectra of polycyclohexene produced using 1a and 1f show the expected three resonances in the *δ* 0.9–2.5 ppm range for the CH_2_ units within the cyclohexane rings (some unreacted cyclohexene is also observed). Farona and Tsonis reported thirty resonances in the *δ* 13.9–35.9 ppm range in the ^13^C NMR spectrum of polycyclohexene, whereas we observe twenty-five clear signals.^46^ Thus, based on the NMR spectra it is clear that homopolymerisation has occurred in both reactions. IR spectroscopy also supports the formation of polycyclohexene using both precatalysts, based on the work of Farona and Tsonis; specifically, the observation of two characteristic bands at 906 and 890 cm^−1^ (lit. 890 and 850 cm^−1^; see SI Fig. S7for the IR spectrum).^22^ A similar conclusion to this previous report can be made here, that the polycyclohexene products are either made up of repeating 1,2-cylohexane units or a combination of 1,2-,1,3-, and/or 1,4-repeating units.^46^

GPC data (Table 2, SI Fig. S19–S22) show that similar, low molecular weight poly(1-hexene) and polycyclohexene are produced using 1a and 1f, which are highly monodisperse. Despite the low molecular weight of polycyclohexene obtained, this is on the higher side of what is previously reported in the literature. The results of DSC thermoanalytical measurements for the two polymers are included in Table 2 and the traces shown in the SI (Fig. S25–S28); although inconclusive with regards to observing side-chain crystallisation, the glass transition temperature obtained for poly(1-hexene), for which previous DSC data have been reported, matches the literature values.^50,51^

**Table 2:** GPC and DSC studies of poly(1-hexene) and polycyclohexene products using precatalysts 1a and 1f, activated by [Ph_3_C][B(C_6_F_5_)_4_]^a^

Monomer	1a				1f			
	*M*_n_ (g mol^−1^)	*M*_w_ (g mol^−1^)	*Đ*	*T*_g_ (°C)	*M*_n_ (g mol^−1^)	*M*_w_ (g mol^−1^)	*Đ*	*T*_g_ (°C)
1-Hexene	4641	4899	1.1	−57.28 (ref. 51)	4518	4761	1.1	−56.24 (ref. 50 and 51)
Cyclohexene	4883	5078	1.0	−50.62	5100	5380	1.0	−49.28

a *M*_n_ = number average molecular weight, *M*_w_ = weight average molecular weight, *Đ* = dispersity determined by GPC (DMF, 0.1% LiBr, 40 °C) relative to polystyrene standards. *T*_g_ = glass transition temperature obtained using DSC.

### Cyclotrimerisation of alkynes

The cyclotrimerisation of alkynes has long been the domain of transition metal catalysts^52–56^ and there has been surprising little work reported using non-d-block catalysts. The first examples of catalytic alkene trimerisation of this type were seen for terminal ethynyl ketones (RCOC≡CH) which trimerise to 1,3,5-benzenes in the presence of dimethylamine *via* enamine intermediates.^57^ In contrast, cyclotrimerisation reactions involving hexachlorodisilane (Cl_6_Si_2_) occur by a stepwise (silicon-centred) radical pathway, producing 1,3,5-benzenes.^58^ The most closely related system to transition metals involves the digermyne precatalyst Tbb–Ge≡Ge–Tbb (Tbb = 2,6-{(Me_3_Si)_2_CH}_2_-C_6_H_3_) which cyclotrimerises terminal alkynes regioselectively to the 1,2,4-isomer and occurs by redox catalysis involving the Ge(ii)/Ge(iv) couple.^59^

**Scheme 4:**
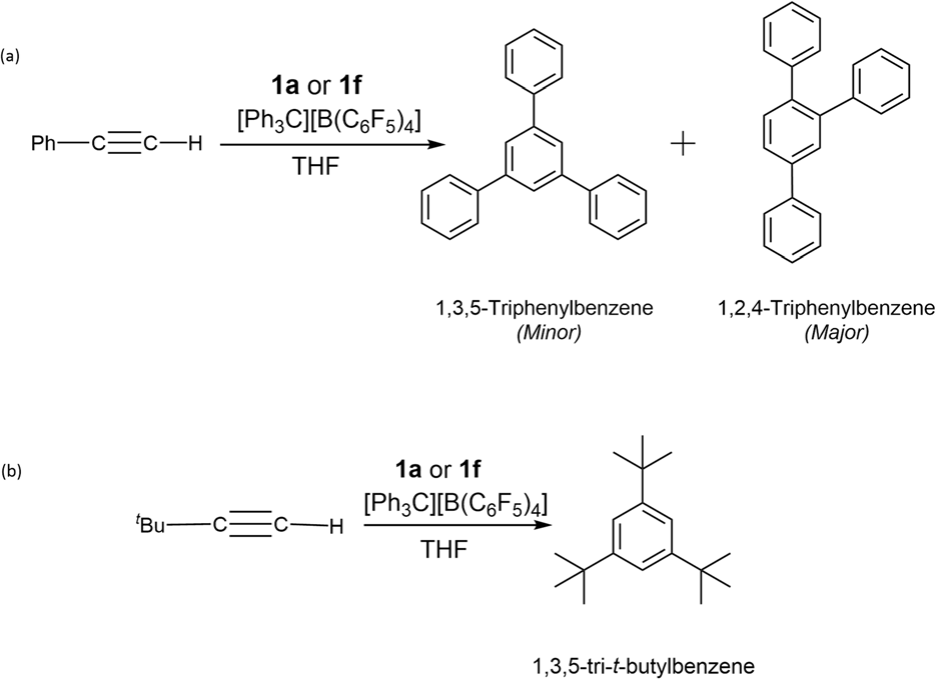
Products of the reactions of (a) phenylacetylene, and (b) *t*-butyl-acetylene with 1a or 1f/[Ph_3_C][B (C_6_F_5_)_4_].

**Fig. 6:**
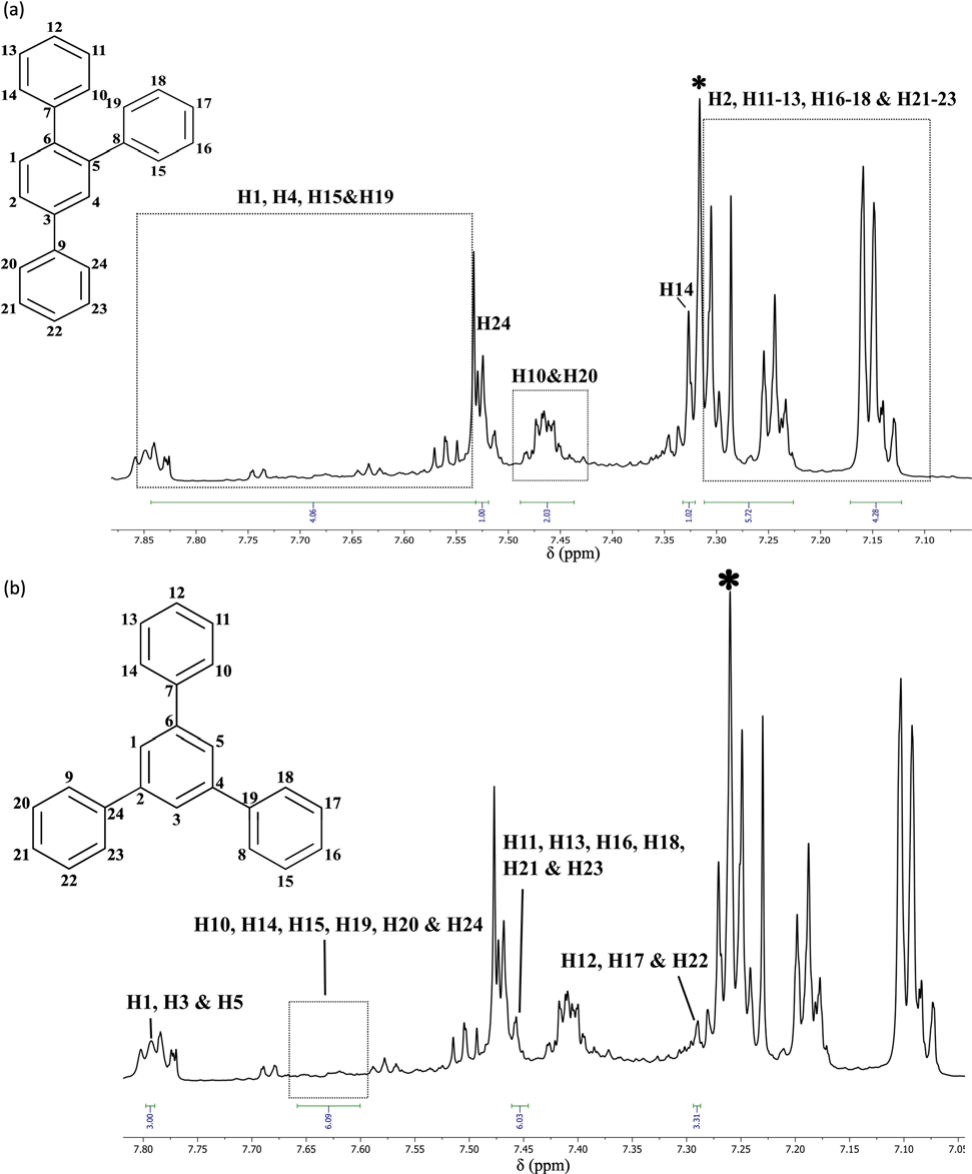
The spectra in (a) and (b) are duplicated here, to allow presentation of the assignments for both isomers more clearly. (a) ^1^H NMR (25 °C, d-chloroform, 700 MHz) spectrum of a mixture of 1,2,4- and 1,3,5-triphenylbenzene prepared using a 0.5 mol% precatalyst loading of 1f and [Ph_3_C][B(C_6_F_5_)_4_] (cation 2f) at room temperature, with the resonances for the 1,2,4-isomer highlighted. (b) The same spectrum with the resonances for 1,3,5-triphenylbenzene highlighted. * d-chloroform.

In our studies of the cyclotrimerisation of terminal alkynes, we again selected precatalysts 1a and 1f and focused initially on phenylacetylene. Cyclotrimerisation to mixtures of (minor) 1,3,5- and (major) 1,2,4-triphenylbenzene^60^ occurred readily using a range of precatalyst loadings (0.5–10 mol%), reaction times (1–24 h) and temperatures (from room temperature to 60 °C) in THF (Scheme 4a). Although the ^1^H NMR spectra (Fig. 6) show some distinct sets of resonances which are consistent with the presence of a combination of trimers in the above samples, these overlap extensively making it difficult to determine the relative amount of the major component. The relative intensities of the peaks, however, confirm that 1,2,4-triphenylbenzene is the major product in our case. Sub-stoichiometric TONs were calculated in the range 10–22 for these reactions.

Further studies show that *t*-butyl acetylene is also cyclotrimerised, this time into 1,3,6-tri-*t*-butylfulvene at 30 °C for 24 h (Scheme 4b).^61–64^ The exclusive formation of the latter was shown by IR spectroscopy, which matches the literature spectrum (ESI Fig. S6).^64^ The presence of 1,3,5-tri-*t*-butyl benzene was discounted on the basis of IR spectroscopy data since it is not consistent with this trimer.^63^

#### Theoretical studies of reaction mechanisms

##### Lewis acidity

The Lewis acid strength of the cation [MeAl(2-py)_2_(µ-Me)AlMe]^+^ [derived from demethylation of [Me_2_Al(2-py)]_2_ (1a)] was initially determined by calculating its fluoride ion affinity (FIA).^65^ Carried out at the ωB97XD/6-311++G(d,p) level,^66–71^ the calculated enthalpy change for the reaction with CF_3_O^−^ (SI), subsequently corrected using the known experimental value,^65^ gave a value of 711 kJ mol^−1^. Compared to 485 kJ mol^−1^ for AlCl_3_ and 637 kJ mol^−1^ for Ph_3_C^+^, this suggests that the capacity of the bridged cation to polymerise alkenes and trimerise phenylacetylene can be attributed to its exceptional Lewis acidity.

**Fig. 7:**
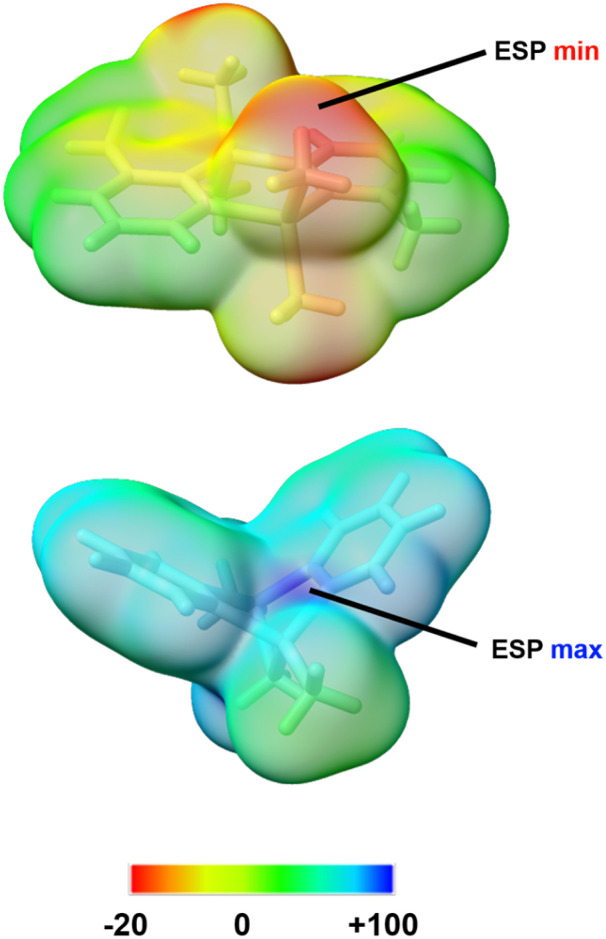
Molecular electrostatic potentials for one of the neutral precatalysts (1b, right) and a catalytically active cationic complex (2a, [MeAl(2-py)_2_(µ-Me)AlMe]^+^ as per our calculations, left) mapped onto the 0.001 a.u. isodensity envelope; the ESP scale is in kcal mol^−1^.

The local distribution of Lewis acidity and basicity in the cation *versus* the neutral precatalyst can be illustrated visually through molecular electrostatic potential (ESP) surface maps (shown for two representative species, precatalyst 1b and cation 2a in Fig. 7).^72^ The ESP for the two species was calculated at the ωB97X-D3/def2-TZVP level of theory,^73–76^ and the value of this rigorously defined physical measure can indicate what an incoming reactant ‘sees’ at the early stages of a covalent interaction.^72^ Notably, the minimum (negative) value is observed on the Lewis basic terminal methyl groups of the neutral precatalyst, consistent with their facile demethylation using Ph_3_C^+^ (Fig. 7, left). Conversely, the maximum (highly positive) value is found at the *σ*-hole site of Al(iii) in the bridged cation, demonstrating the extraordinary Lewis acidity at that location.

Complementary to the ESP surface analysis, local nucleophilicity and electrophilicity can be probed using theory-derived measures such as the average local ionisation energy (ALIE) and the local electron attachment energy (LEAE), respectively.^77–79^ Their mappings on the surfaces of the two representative molecules (at the same level of theory as the ESP) lead to similar conclusions and highlight, in particular, the reactivity of the Al(iii) *σ*-hole in the bridged cation (SI Fig. S29 and S30).^80–82^ The reactive nature of the activated alkyl-bridged cations, as illustrated by the FIA calculations and quantitative molecular surface analysis, presumably adds to the practical difficulty of isolating these species for crystallographic characterisation.

##### Mechanism of Polymerisation

**Fig. 8:**
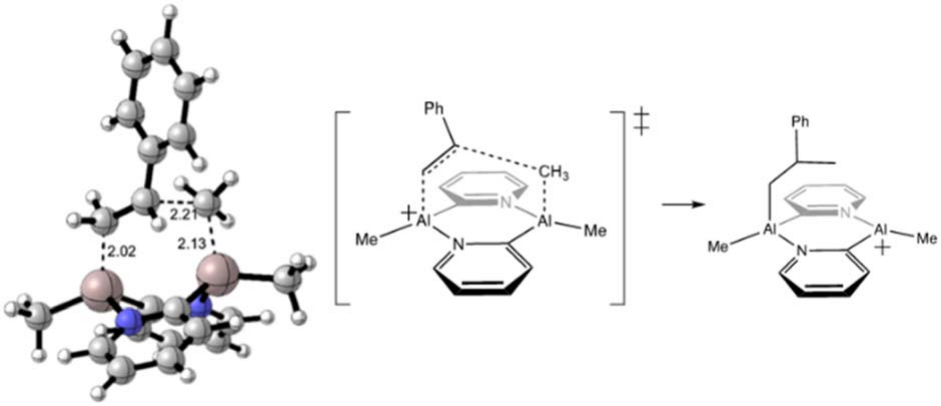
Initially calculated transition state (Δ*G*^‡^ = 150.2 kJ mol^−1^) representing the “switching” of the monomer between the Al atoms.

DFT calculations were conducted at the ωB97XD/6-311++G(d,p)//B3LYP-D3/6-31G(d) level^65^ of theory to assess whether the initial mechanism of polymerisation we had proposed (see Scheme 2) is viable, using styrene as the monomer. Due to the large conformational space associated with torsional freedom in ^i^Bu groups, they were replaced by methyl groups to reduce the computational cost. Further, the substituents on the pyridine ring were replaced by an H-atom for initial studies into the mechanistic pathway since they were deemed to be unlikely to participate significantly. Initial calculations following a concerted carboalumination reaction showed that the barrier to this mechanism was too high (*ca.* 150 kJ mol^−1^). A transannular carboalumination also had an excessively high energy transition state (Δ*G*^‡^ = 150.2 kJ mol^−1^) (Fig. 8).

**Fig. 9:**
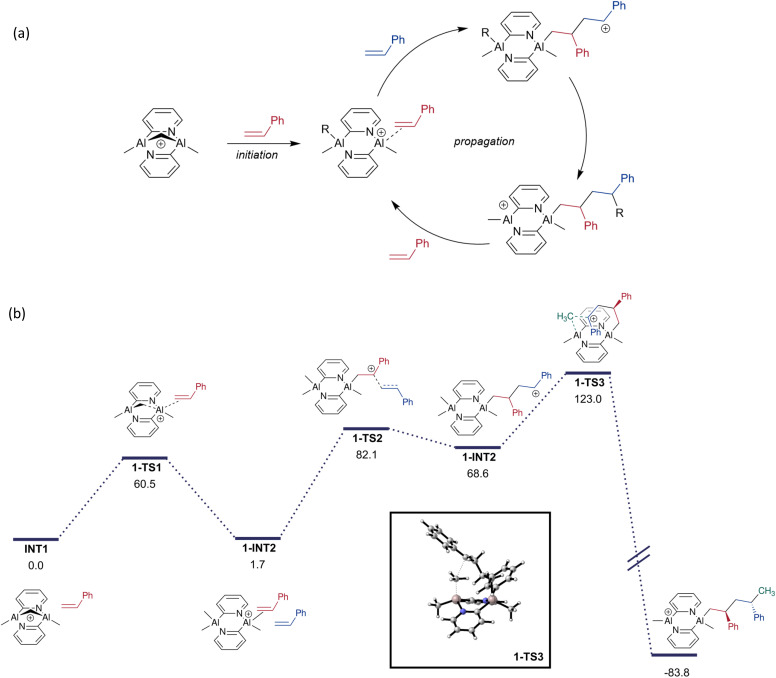
(a) Viable mechanism involving dimer transfer, and (b) energy profile of the dimer transfer-based polymerisation mechanism (Gibbs free energies in kJ mol^−1^). The TS for the S_E_2 (back) reaction is highlighted in the box.

Polymer chain growth by dimer transfer was therefore considered as an alternative mechanism (Fig. 9a). Following activation of one monomeric unit by the catalyst, the π (C=C) bond of another styrene monomer attacks the Al-bonded alkene. The resulting benzylic carbocation 1-INT2 is now part of a 4-carbon tether that can access the axial methyl group on the second Al atom. This participates in a *S*_E_2 reaction that migrates the methyl group onto the tether and creates a new highly Lewis acidic site that can activate another styrene molecule. The cycle continues by further dimerisation of styrene at the Al centre, generating another tethered benzylic carbocation that participates in an *S*_E_2 reaction to elongate the growing chain by two monomeric styrene units.

The electronic energy profile of the reaction (and the enthalpy profile) of the suggested mechanism has sufficiently low barriers to be feasible at room temperature (SI). However, significant loss in translational entropy during the polymerisation raises the free energy of intermediates, with each styrene association having an entropic cost of around 167 J K^−1^ mol^−1^ (50 kJ mol^−1^ of free energy at room temperature). A free-energy adjusted profile (Fig. 9b) has the largest barrier at 121 kJ mol^−1^ above the resting state catalyst. Despite this, styrene will be present in large excess, it is therefore possible that this pathway may still be operative given the enthalpic favourability.

**Fig. 10:**
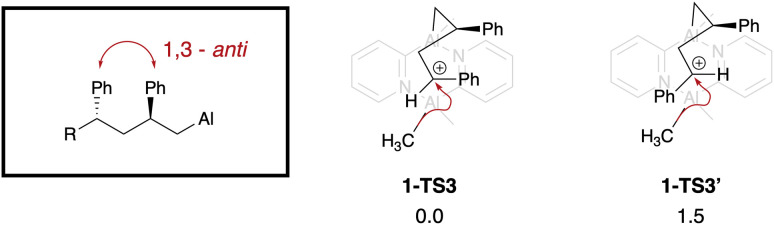
Stereochemistry of the polymer backbone as dictated by the addition of a second styrene monomer. The resulting *anti* arrangement in the polymer chain is highlighted. Energies are relative electronic energies in in kJ mol^−1^.

We next explored the origin of the experimentally observed stereocontrol. The first stereocentre to be formed during the polymerisation of styrene occurs during the addition of the second styrene monomer. The relative stereochemistry between the two phenyl groups is then dictated by the geometry of the *S*_E_2 step. The energies of the diastereomeric transition states (1-TS3 and 1-TS3′) differ by 1.5 kJ mol^−1^ (Fig. 10) and the experimentally observed *anti* stereochemistry is favoured.

**Fig. 11:**
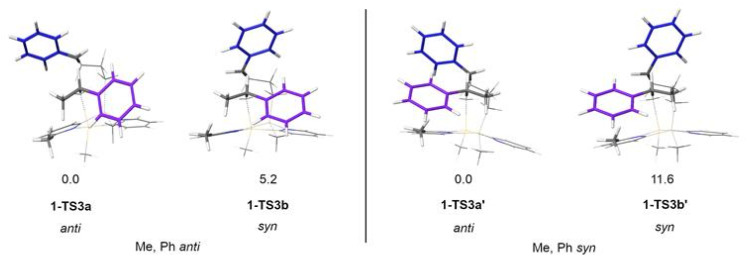
The four isomeric model transition states studied. Values given are relative Gibbs free energies in kJ mol^−1^. Absolute stereochemistry is labelled according to the relation between Me on catalyst and Ph on chain (purple) in the conformation when H projects into other chain.

A model system was constructed to study the stereochemical outcome of the *S*_E_2 step, this time featuring the Me substituent in the 2-position of the pyridine. The (styrene, styrene) chain was replaced with an (ethene, styrene) chain to maintain the benzylic carbocation at the reacting centre. The other chain was constructed with a Me group capping the styrene unit in place of another styrene monomer. Calculations showed only conformers with the H atom projecting towards chain are sufficiently low energy (SI). This left four isomeric transition states to be considered (outlined in Fig. 11). For either absolute stereochemistry, the *anti* TS is favoured over the *syn*.

At 298 K, the energy of the *syn* structure 1-TS3b is 5.2 kJ mol^−1^ higher than that of the most stable *anti* arrangement 1-TS3a, which corresponds to a 9 : 1 preference for the *anti*-stereochemistry; this means that the *anti* relationship of the Ph groups will be favoured in the polymer chain, promoting the formation of the syndiotactic polymer as observed experimentally. The extent of the stereoselectivity is predicted to be higher in the isomeric 1-TS3a′.

**Fig. 12:**
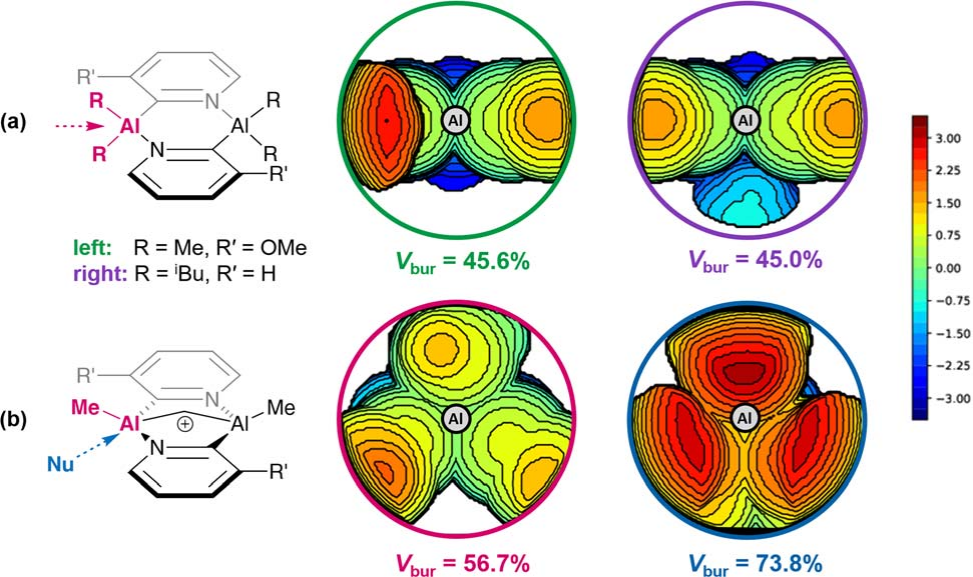
Topographic steric maps showing the steric pressure exerted on each aluminium centre: (a) by the ring system in the neutral precatalysts 1c and 1d (left and right, respectively; viewed on the plane of the ring from the direction of the alkyl groups); (b) by the ring system in the active cationic catalyst 2a (viewed from the direction of the terminal methyl) (left) or by the entire ligand scaffold (including terminal alkyls, viewed from the direction of the incoming substrate) (right). The scale of the isocontours of altitude from the Al centre range between ± 3.5 Å.

The steric properties of the system can be examined further using topographic steric maps, which are commonly used to rationalise the behaviour of catalytic pockets.^83,84^ Two neutral systems (precatalysts 1c and 1d), representative of the start of the propagation step, were selected for their differing R and R′ groups (Fig. 12a). The examination of the steric profile on each aluminium (excluding the growing chain) reveals the local effect of the 2-pyridyl substituents (Fig. 12a, left), while the overall hindrance from the ring system is not highly variable—in line with our considerations above for the *S*_E_2 step. In contrast, the bridged cationic species (as exemplified by cation 2a, Fig. 12b), experiences a much more pronounced steric pressure from the ring system. Such an effect, especially along the direction of the approaching alkene, can have a dramatic impact in the energetics of the initiation step; previous studies have investigated the relation between the percentage of buried volume (*V*_bur_) and Lewis acidity.^85^

##### Mechanism of cyclotrimerisation

**Fig. 13:**
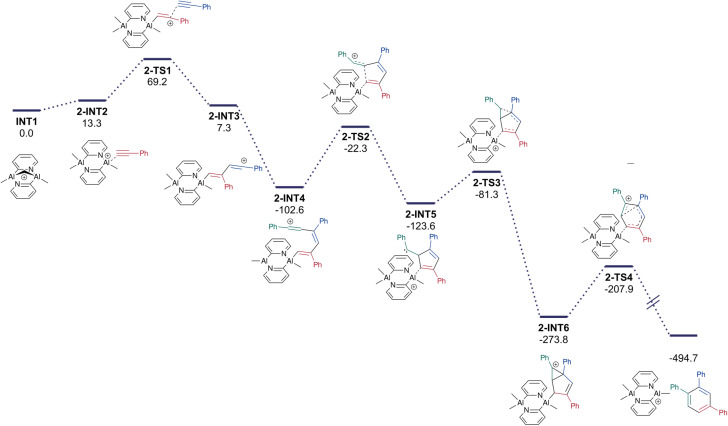
Proposed reaction mechanism and energy profile diagram of the calculated trimerisation reaction pathway (Gibbs free energies in kJ mol^−1^).

The cyclotrimerisation of phenylacetylene into triphenyl-benzene was subsequently investigated (Fig. 13a). The rigidity of the sp^2^ carbon-containing tether would preclude the *S*_E_2 reaction suggested for styrene polymerisation, so the action of the complex as a Lewis acid on one Al site was considered, starting with the addition of the phenylacetylene to the rest state of the catalyst. This addition was found to be electronically barrierless.

Addition of a second alkyne gives diene 2-INT3 which then undergoes another barrierless addition (SI) to form 2-INT4. Cyclisation *via* a five-membered ring forms a benzylic carbene 2-INT5. Addition of the carbene to the π bond results in cyclopropane 2-INT6 which undergoes a ring-opening to give the final 1,2,4-subsituted aromatic product (Fig. 13). An alternative carbene addition to the Al-bonded C atom, on the pathway to the 1,3,5-product, resulted in a higher energy TS (Δ*E* = 4 kJ mol^−1^; SI), consistent with the experimental results. The carbene intermediate may also undergo a 1,2 H shift to form the fulvene product. It is likely that steric crowding makes this process dominant over the carbene addition when *t*-butylacetylene is the substrate used (SI).

## Conclusions

In this study we have shown that dimers [R_2_Al(2-py′)]_2_ (1) are precatalysts for the polymerisation of alkenes and for the cyclotrimerisation of alkynes. Although only low molecular weight polymers are produced from alkenes, syndioselectivity is observed especially where sterically bulky Al-bonded groups are present. To the best of our knowledge this is the first alkene polymerisation catalyst based on Al(iii) where stereoselectivity has been observed. In addition, the observed trimerisation of alkynes is not only a rare example involving non-transition element catalysis of this type but is also the first example involving aluminium (or any fully metallic main group element). DFT calculations trace this reactivity to the high Lewis acidity of the catalytically active cations, which is greater than that of Ph_3_C^+^. Theoretical studies also indicate that a co-operative polymerisation mechanism involving dimer transfer is viable, in which both Al(iii) centres of the proposed catalytic intermediates are involved. This mechanism can also explain the syndioselectivity of the resulting polymers. A related mechanism also explains the cyclotrimerisation of alkynes.

Above all, this work shows the potential promise of static catalysis based on Al(iii) in a broad range of organic transformations. On this basis, we are expanding our studies to explore other transition metal-mimetic systems based on aluminium.

## Author contributions

DC did all of the synthetic work, collated experimental data, and supplied samples for GPC analysis. RD performed DFT calculations pertaining to the mechanism. AT performed NMR measurements and the calculations for the qualitative molecular surface analyses and steric maps. NSP did the GPC measurements. OAS, JMG and DSW supervised the project. DSW and JMG devised the idea. All authors contributed to the writing of the initial manuscript, which was revised by DC, AT and DSW. All authors read and approved the final version.

## Conflicts of interest

There are no conflicts to declare.

## Supplementary Material

SC-017-D5SC07624B-s001

## Data Availability

Data is available in the supplementary information (SI) that accompanies this paper. DFT output files are available at https://doi.org/10.17863/CAM.121268. The supplementary information includes the synthetic procedures, IR and NMR spectra, GPC and DSC data, and full details of DFT calculations; see DOI: https://doi.org/10.1039/d5sc07624b.
