# Supplementary material for: Oligomerisation and stereoselective polymerisation of alkenes and alkynes using pyridyl-based Al(iii) catalysts
Source: Chem Sci. 2026 Jan 21;17(12):5953–64. doi: 10.1039/d5sc07624b (PMC12871876; doi:10.1039/d5sc07624b)
Supplement: SC-017-D5SC07624B-s001 [file SC-017-D5SC07624B-s001.pdf]

Supplementary Information (SI) for Chemical Science.
This journal is © The Royal Society of Chemistry 2026

Oligomerisation and Stereoselective Polymerisation of Alkenes and Alkynes Using Pyridyl-based Al(III) Catalysts

Dipanjana Choudhury,[ǂ,a] Richard Danylyuk,[ǂ,a] Alexandros Terzopoulos,[a] Natalie S. Potter,[a] Oren A. Scherman,*[,a] Jonathan M. Goodman*[a] and Dominic S. Wright*[a]

[a]Yusuf Hamied Department of Chemistry, University of Cambridge, Lensfield Rd, Cambridge CB2 1EW (U.K.); e-mail: oas23@cam.ac.uk, mg11@cam.ac.uk, dsw1000@cam.ac.uk.

[ǂ] Both authors should be considered first author of this paper.

Table of Contents

Experimental details 3
Additional NMR spectra 12
IR spectra 14
SEC traces 15
DSC thermal analysis 23
Computational methods 29
Fluoride ion affinities (FIA) 30
Qualitative molecular surface analysis 31
Stereochemistry of chain transfer 32
Enthalpy profile for polymerisation 32
NBO calculations 33
Scans for barrierless steps 34
Ring closing 35
Alternative regioselectivity 38
Geometry of alkene 39
Elimination pathway 40

Experimental details

All synthetic processes were performed using standard Schlenk techniques under a dry nitrogen atmosphere using oven-dried glassware. Starting materials were acquired from commercial suppliers (Aldrich, Acros Organics, Alfa Aesar, Manchester Organics and Fluorochem) and used as received, unless otherwise specified. Toluene and *n*-hexane were dried using sodium and distilled under nitrogen, acetonitrile and DCM were dried over calcium hydride and stored over 4 Å molecular sieves, and THF was distilled over sodium/benzophenone. Low temperatures in syntheses were attained using a dry ice/acetone (−78 °C) or a dry ice/ethylene glycol (−15 °C) bath. All air-sensitive materials were handled in a nitrogen-filled glovebox (Saffron type α). ^{1}H, ^{13}C, ^{27}Al, ^{11}B, and ^{19}F NMR spectra were recorded on a Bruker Avance III HD 500 MHz Smart Probe spectrometer; higher-resolution ^{1}H (with dynamic flip angle control based on T_1 relaxation) and ^{13}C NMR spectra were recorded on a Bruker Avance III HD 700 MHz TXO Cryoprobe spectrometer. For ^{1}H and ^{13}C NMR, chemical shifts are internally referenced to the deuterated solvent and calculated relative to TMS. The internal reference standard used for ^{27}Al NMR was $AlCl_3 \cdot 6H_2O$, for ^{11}B NMR $BF_3 \cdot OEt_2$ in $CDCl_3$, and for ^{19}F NMR neat $CFCl_3$. Single-Crystal X-ray diffraction data were obtained using either a Bruker D8-QUEST PHOTON-100 diffractometer (Cu-Kα, λ = 1.54178 Å) or a Nonis KappaCCD diffractometer (Mo-Kα, λ = 0.71073 Å). Polymer molecular weights and molecular weight distributions were assessed using a DMF GPC method. DMF GPC was performed on a series of three SUPREMA (100Å, 3000Å and 3000Å) columns with an SPD-M20A prominence diode array detector and RID-20A refractive index detector (both Shimadzu) calibrated in relation to polystyrene standards. Samples were filtered through a 0.2 µm PTFE filters before injection. The GPC eluent was HPLC-grade DMF containing 1g/L LiBr at a flow rate of 1.0 ml/min. The small molecule LCMS analysis was conducted on the mass spectrometer Xevo G2-S coupled with UPLC system. The column used for the LC was Acquity UPLC BEHC18 with dimensions 2.1 mm × 50 mm. The gradient for the method was: Time 0 min, Flow rate 0.2 mL/min for composition 95% A solution (Water with 0.1 % Formic acid) and 5% B (Acetonitrile), Time 1.00, 95% A and 5% B, Time 5 min, 0% A and 100% B, Time 6 min 0% A and 100% B and time 7 min 95% A and 5% B. The data were processed by MassLynx software that controls LCMS analysis and runs Xevo. Fourier transform infrared spectroscopy (FTIR) spectra were collected on a Thermo Scientific Nicolet iS50 FTIR spectrometer (ATR mode) in the range of 400 to 4000 cm^{-1}. Omnic software was used for analysis. Differential scanning calorimetry (DSC) was conducted on a Discovery 2500

instrument manufactured by TA Instruments (Waters). For polystyrene samples, the measurements were conducted in the Modulated Conventional mode with a modulated temperature amplitude of 1 °C, a modulation period of 120 s, ramp rate of 5 °C/min from 0 °C to 300 °C. For poly(1-hexene) and polycyclohexene samples, the measurements were conducted in the Modulated Conventional mode with a modulated temperature amplitude of 1 °C, a modulation period of 120 s, ramp rate of 5 °C/min from −90 °C to 200 °C.

Polymerisation of styrene (NMR-scale): Dimer **1a** (56.7 mg, 0.211 mmol) and $[Ph_3C][B(C_6F_5)_4]$ (194 mg, 0.211 mmol) were dissolved in d_8-toluene (~ 0.7 mL) in a J Young NMR tube. Styrene was added according to the desired catalytic loading of cation **2a** (10 mol%: 2.1 mmol, 0.22 mL; 5 mol%: 4.2 mmol, 0.48 mL; 2.5 mol%: 8.4 mmol, 0.96 mL) and the reaction was maintained at 100 °C overnight under a N_2 atmosphere. For the 0.5 mol% catalytic loading, the reaction was conducted at a 0.105 mmol-scale bearing the overall volume of the NMR tube in mind (dimer **1a**: 28.38 mg; $[Ph_3C][B(C_6F_5)_4]$: 96.85 mg; styrene: 21 mmol, 2.4 mL). *In-situ* ^{1}H NMR (500 MHz, 25 °C, d_8-toluene): δ 7.02–7.13 (mult, 3H, H(2)–H(4)), 6.45–6.94 (mult, bd, 2H, H(1) & H(5)), 0.98–1.84 (mult, bd, 2H, H(7) & H(8)) ppm. ^{13}C NMR (126 MHz, 25 °C, d_8-toluene): δ 149.1 (Ph), 145–146.8 (bd, C(6)), 137 (Ph), 128.0 (C(1) & C(5)), 127.9 (C(2) & C(4)), 125.9 (C(3)), 40.6 (C(7)), 41.5–47 (C(8)) ppm. ^{11}B NMR (25 °C, d_8-toluene, 160 MHz): δ −16 ppm. ^{19}F NMR (25 °C, d_8-toluene, 471 MHz): δ −132.3, −163.1, −167.1 ppm. GPC: M_n = 1689 g mol^{-1}, M_w = 3595 g mol^{-1}, $Đ$ = 2.1.

*Polymerisation of styrene using dimer **1a** (cation **2a**)*: Dimer **1a** (28.38 mg, 0.105 mmol) and $[Ph_3C][B(C_6F_5)_4]$ (96.85 mg, 0.105 mmol) were dissolved in toluene (~ 1–2 mL). Styrene was added dropwise according to the desired catalytic loading of cation **2a** (10 mol%: 1.05 mmol, 0.12 mL; 5 mol%: 2.1 mmol, 0.24 mL; 2.5 mol%: 4.2 mmol, 0.48 mL; 0.5 mol%: 21 mmol, 2.4 mL) and the reaction was stirred for one hour at room temperature. The reaction was quenched using a few drops of dry MeOH. The solvent was taken off *in vacuo*, leaving behind a “plastic”-like solid. The procedure was repeated at −15 °C. ^{1}H NMR (500 MHz, 25 °C, d_8-toluene): δ 7.01–7.16 (mult, 3H, H(2)–H(4)), 6.43–6.96 (mult, bd, 2H, H(1) & H(5)), 0.97–1.82 (mult, bd, 2H, H(7) & H(8)) ppm. ^{13}C NMR (126 MHz, 25 °C, d_8-toluene): δ 144.3–146.8 (bd, C(6)), 137 (Ph), 128.0 (C(1) & C(5)), 127.7 (C(2) & C(4)), 125.2 (C(3)), 41.3–46.7 (C(7)), 40.6 (C(8)) ppm. GPC: M_n = 1689 g mol^{-1}, M_w = 3595 g mol^{-1}, $Đ$ = 2.1 (2.5 mol%, RT); M_n =

2191 g mol^{-1}, M_w = 6514 g mol^{-1}, $Đ$ = 2.9 (0.5 mol%, RT); M_n = 5142 g mol^{-1}, M_w = 6030 g mol^{-1}, $Đ$ = 1.2 (2.5 mol%, −15 °C); M_n = 5103 g mol^{-1}, M_w = 8687 g mol^{-1}, $Đ$ = 1.7 (0.5 mol%, −15 °C).

*Polymerisation of styrene using dimer **1e** (cation **2e**)*: Dimer **1e** (49 mg, 0.105 mmol) and $[Ph_3C][B(C_6F_5)_4]$ (96.85 mg, 0.105 mmol) were dissolved in toluene (~ 1–2 mL). Styrene was added dropwise according to the desired catalytic loading of cation **2e** (10 mol%: 1.05 mmol, 0.12 mL; 5 mol%: 2.1 mmol, 0.24 mL; 2.5 mol%: 4.2 mmol, 0.48 mL; 0.5 mol%: 21 mmol, 2.4 mL) and the reaction was stirred for 48 h (1 h for the 10 mol% case) at −15 °C. The reaction was quenched using a few drops of dry MeOH. The solvent was taken off *in vacuo*, leaving behind a "plastic"-like solid in combination with a more "powdery" solid. Yield 898.8 mg, 41%. TON: 82. ^{1}H NMR (500 MHz, 25 °C, d_8-toluene): δ 7.01–7.15 (mult, 3H, H(2)–H(4)), 6.45–6.95 (mult, bd, 2H, H(1) & H(5)), 0.97–1.73 (mult, bd, 2H, H(7) & H(8)) ppm. ^{13}C NMR (126 MHz, 25 °C, d_8-toluene): δ 144.4–146.8 (bd, C(6), atactic), 143.9 (C(6), syndiotactic) 137 (Ph), 128.0 (C(1) & C(5)), 127.7 (C(2) & C(4)), 125.2 (C(3)), 40.2–46.8 (C(7) & C(8), syndiotactic+atactic) ppm. GPC: M_n = 2613 g mol^{-1}, M_w = 3195 g mol^{-1}, $Đ$ = 1.2 (10 mol%, −15 °C); M_n = 2045 g mol^{-1}, M_w = 5307 g mol^{-1}, $Đ$ = 2.6 (5 mol%, −15 °C); M_n = 1411 g mol^{-1}, M_w = 2959 g mol^{-1}, $Đ$ = 2.1 (2.5 mol%, −15 °C); M_n = 5123 g mol^{-1}, M_w = 10376 g mol^{-1}, $Đ$ = 2.0 (0.5 mol%, −15 °C). IR (cm^{-1}): 3057, 3028, 2918, 2844, 1603, 1492, 1451, 1258, 1079, 1025, 759, 696, 538.

For 2.5 mol% loading, ^{13}C NMR (126 MHz, 25 °C, d_8-toluene): δ 146.9 (C(6), isotactic), 146.5 (C(6), isotactic) 144.8–146.3 (bd, C(6), atactic), 143.9 (C(6), syndiotactic), 137 (Ph), 128.0–128.1 (C(1) & C(5)), 127.7 (C(2) & C(4)), 125.2–126.1 (C(3)), 48.5 (MeOH), 44.2 (C(8), syndiotactic), 40.1–41.2, 37.44 (C(7)) ppm.

*Polymerisation of styrene using dimer **1b** (cation **2b**)*: Dimer **1b** (31.33 mg, 0.105 mmol) and $[Ph_3C][B(C_6F_5)_4]$ (96.85 mg, 0.105 mmol) were dissolved in toluene (~ 1–2 mL) to achieve a catalytic loading of 0.5 mol%. Styrene was added dropwise (21 mmol, 2.4 mL) and the reaction was stirred for 1 h at 0 °C. The reaction was quenched using a few drops of dry MeOH. The solvent was taken off *in vacuo*, leaving behind a mass of "plastic"-like solid. Yield 1795.3 mg, 82%. TON: 164. ^{1}H NMR (500 MHz, 25 °C, d-chloroform): δ 7.29–7.51 (mult, 3H, H(2)–H(4)), 6.64–6.99 (mult, bd, 2H, H(1) & H(5)), 2.59 (py substituent, CH_3), 1.25–2.28 (mult, bd,

2H, H(7) & H(8)) ppm. ^{13}C NMR (126 MHz, 25 °C, d_8-toluene): δ 145.0–146.8 (bd, C(6), atactic), 137.94 (Ph), 128.2 (C(1) & C(5)), 127.8 (C(2) & C(4)), 125.5 (C(3)), 40.3–46.1 (C(7) & C(8), atactic), 21.63 (py substituent, CH_3) ppm. GPC: M_n = 2118 g mol^{-1}, M_w = 10740 g mol^{-1}, $Đ$ = 5.0.

*Polymerisation of styrene using dimer **1c** (cation **2c**)*: Dimer **1c** (31.33 mg, 0.105 mmol) and $[Ph_3C][B(C_6F_5)_4]$ (96.85 mg, 0.105 mmol) were dissolved in toluene (~ 1–2 mL) to achieve a catalytic loading of 0.5 mol%. Styrene was added dropwise (21 mmol, 2.4 mL) and the reaction was stirred for 1 h at 0 °C. The reaction was quenched using a few drops of dry MeOH. The solvent was taken off *in vacuo*, leaving behind a mass of "plastic"-like solid (Figure S1). Yield 1446.2 mg. ^{1}H NMR (500 MHz, 25 °C, d-chloroform): δ 7.31–7.54 (mult, 3H, H(2)–H(4)), 6.79–7.13 (mult, bd, 2H, H(1) & H(5)), 2.70 (py substituent, OCH_3), 1.39–2.46 (mult, bd, 2H, H(7) & H(8)) ppm. ^{13}C NMR (126 MHz, 25 °C, d-chloroform): δ 145.2–146.7 (bd, C(6), atactic), 138.0 (Ph), 128.4 (C(1) & C(5)), 127.9 (C(2) & C(4)), 126.0 (C(3)), 40.5–47.3 (C(7) & C(8), atactic), 21.8 (py substituent, OCH_3) ppm. GPC: M_n = 3515 g mol^{-1}, M_w = 13602 g mol^{-1}, $Đ$ = 3.9. IR (cm^{-1}): 3059, 3024, 2921, 2845, 1601, 1492, 1452, 1079, 1027, 756, 695, 537.

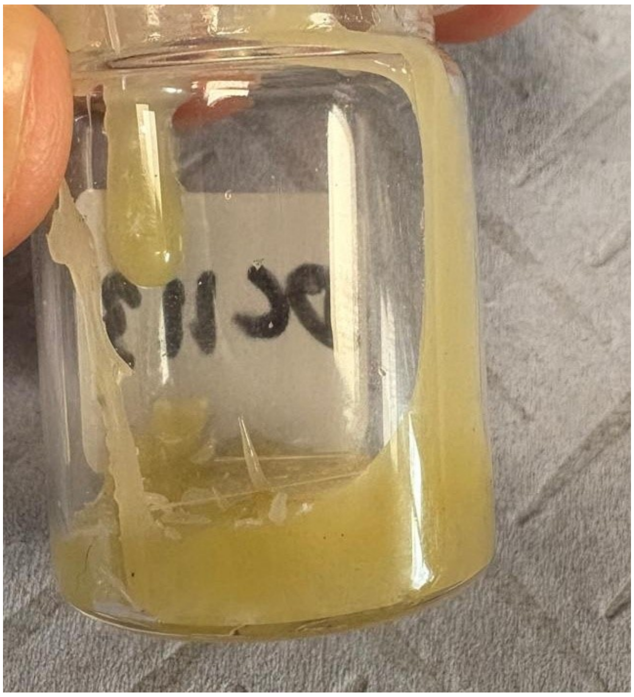

Figure S1. Image of atactic polymeric product obtained using cation **2c**.

*Polymerisation of styrene using dimer **1f** (cation **2f**)*: Dimer **1f** (52.36 mg, 0.105 mmol) and $[Ph_3C][B(C_6F_5)_4]$ (96.85 mg, 0.105 mmol) were dissolved in toluene (~ 1–2 mL) to achieve a catalytic loading of 0.5 mol%. Styrene was added dropwise (21 mmol, 2.4 mL) and the reaction was stirred for 1 h at 0 °C. The reaction was quenched using a few drops of dry MeOH. The solvent was taken off *in vacuo*, leaving behind a mass of "plastic"-like solid in combination with a more "powdery" solid (Figure S2). Yield 1070.7 mg, 50%. TON: 100. ^{1}H NMR (500 MHz, 25 °C, d-chloroform): δ 7.33–7.53 (mult, 3H, H(2)–H(4)), 6.72–7.13 (mult, bd, 2H, H(1) & H(5)), 2.65 (py substituent, OCH_3), 1.32–2.59 (mult, bd, 2H, H(7) & H(8)) ppm. ^{13}C NMR (126 MHz, 25 °C, d-chloroform): δ 144.9–146.8 (bd, C(6), atactic), 143.9 (C(6), syndiotactic), 138.0 (Ph), 128.5 (C(1) & C(5)), 128.0 (C(2) & C(4)), 125.9 (C(3)), 40.6–47.0 (C(7) & C(8), syndiotactic+atactic), 21.7 (py substituent, OCH_3) ppm. GPC: M_n = 1961 g mol^{-1}, M_w = 5929 g mol^{-1}, $Đ$ = 3.0. IR (cm^{-1}): 3061, 3025, 2923, 2847, 1601, 1493, 1452, 1260, 1081, 1025, 756, 695, 539.

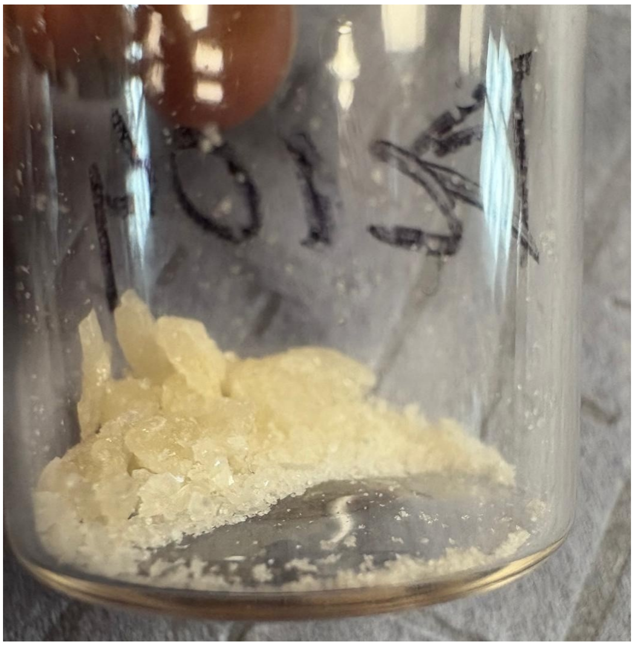

Figure S2. Image of syndiotactic + atactic polymeric product obtained using cation **2f**.

Control reaction for polymerisation using only $[Ph_3C][B(C_6F_5)_4]$: $[Ph_3C][B(C_6F_5)_4]$ (96.85 mg, 0.105 mmol) was dissolved in d_8-toluene (~ 0.7 mL) in a J Young NMR tube. Styrene was added to achieve a catalytic loading of 10 mol% (1.05 mmol, 0.12 mL) and the reaction was sonicated for 1 h at at room temperature. The reaction was quenched using a few drops of dry MeOH. All volatiles were removed *in vacuo* 0 °C, leaving behind a mass of "plastic"-like solid. The ^{13}C NMR spectrum (126 MHz, 25 °C, d-chloroform) is shown in Figure S5 (with resonances for atactic polystyrene highlighted). GPC: M_n= 2464, $Đ$ = 2.3.

*Trimerisation of phenylacetylene using dimer **1a** (cation **2a**)*: Dimer **1a** (28.38 mg, 0.105 mmol) and $[Ph_3C][B(C_6F_5)_4]$ (96.85 mg, 0.105 mmol) was dissolved in 2 mL of THF to achieve a catalytic loading of 0.5 mol%. Phenylacetylene was added (21 mmol, 2.31 mL) and the reaction was stirred at room temperature for 3 h. The resulting brown solution was quenched with excess MeOH; the solvents were removed *in vacuo*, and the product was a brownish-white solid. Yield 178.0 mg, 8.3%, TON: 17

1,2,4-triphenylbenzene:

^{1}H NMR (500 MHz, 25 °C, d-chloroform): δ 7.54–7.68 (mult, bd, 4H, H(1), H(4), H(15) & H(19)), 7.53 (s, 1H, H(24)), 7.45–7.50 (mult, bd, 2H, H(10) & H(20), 7.37 (mult, bd, 1H, H(14)), 7.16–7.31 (mult, H(2), H(11)–H(13), H(16)–H(18) & H(21)–H(23)) ppm. ^{13}C NMR (126 MHz, 25 °C, d-chloroform): δ 143.8 (C(7) & C(8)), 142.5 (C(9)), 133.7 (C(3) & C(5)), 132.0 (C(6)), 129.4 (C(11), C(13), C(16), C(18), C(21) & C(23)), 128.7 (C(1)), 128.2 (C(10), C(14), C(15), C(19), C(20) & C(24)), 127.8 (C(12), C(17) & C(22)), 126.8 (C(4)), 126.2 (C(2)) ppm.

1,3,5-triphenylbenzene:

^{1}H NMR (500 MHz, 25 °C, d-chloroform): δ 7.84 (s, 3H, H(1), H(3) & H(5)), 7.75 (mult, 6H, H(10), H(14), H(15), H(19), H(20), H(24)), 7.52 (mult, bd, 6H, H(11), H(13), H(16), H(18), H(21) & H(23), 7.37 (mult, bd, 1H, H(14)), 7.37–7.46 (mult, bd, H(12), H(17) & H(22) ppm. ^{13}C NMR (126 MHz, 25 °C, d-chloroform): δ 140.7 (C(7)–C(9)), 137.0 (C(2),C(4) & C(6)), 129.9 (C(11), C(13), C(16), C(18), C(21) & C(23)), 127.6 (C(10), C(14), C(15), C(19), C(20) & C(24)), 127.2 (C(12), C(17) & C(22)), 125.9 (C(1), C(3) & C(5)) ppm.

IR(cm^{-1}): 3062, 3021, 2964, 1642, 1596, 1512, 1460, 1273, 1081, 1053, 976, 755, 699, 571. MS $[M+H]^{+}$: found: 307.1477, theoretical: 307.1487.

*Trimerisation of phenylacetylene using dimer **1f** (cation **2f**)*: Dimer **1f** (52.36 mg, 0.105 mmol) and $[Ph_3C][B(C_6F_5)_4]$ (96.85 mg, 0.105 mmol) was dissolved in 2 mL of THF to achieve a range of catalytic loadings (Table 6.1). Phenylacetylene was added and a variety of reaction conditions were used, which have been tabulated below (Table 6.1). In all cases, the resulting brown solution was quenched with excess MeOH; the solvents were removed *in vacuo*, and the product was a brownish-white solid.

Table 6.1. Experimental conditions, yield and TON for the trimerisation of phenylacetylene.

Loading (mol%)	Amount of phenylacetylene (mL)	Time (h)	Temperature (°C)	Yield (mg)	Percentage yield (%)	TON
10	0.12	2	30	104.5	97.4	10
5	0.23	24	30	117.6	55	11
2.5	0.46	5	60	171.6	40	16
0.5	2.31	3	RT	240.4	11	22

1,2,4-triphenylbenzene:

^{1}H NMR (500 MHz, 25 °C, d-chloroform): δ 7.53–7.81 (mult, bd, 4H, H(1), H(4), H(15) & H(19)), 7.52 (s, 1H, H(24)), 7.42–7.50 (mult, bd, 2H, H(10) & H(20), 7.36 (mult, bd, 1H, H(14)), 7.14–7.29 (mult, H(2), H(11)–H(13), H(16)–H(18) & H(21)–H(23)) ppm. ^{13}C NMR (126 MHz, 25 °C, d-chloroform): δ 143.8 (C(7) & C(8)), 142.5 (C(9)), 132.0 (C(6)), 129.4 (C(11), C(13), C(16), C(18), C(21) & C(23)), 128.7 (C(1)), 128.2 (C(10), C(14), C(15), C(19), C(20) & C(24)), 126.2 (C(2)) ppm. Note that some signals could not be observed in this case.

1,3,5-triphenylbenzene:

^{1}H NMR (500 MHz, 25 °C, d-chloroform): δ 7.81 (s, 3H, H(1), H(3) & H(5)), 7.73 (mult, 6H, H(10), H(14), H(15), H(19), H(20), H(24)), 7.51 (mult, bd, 6H, H(11), H(13), H(16), H(18), H(21) & H(23), 7.39–7.42 (mult, bd, H(12), H(17) & H(22) ppm. Note that the sample was too dilute for the ^{13}C NMR signals to be differentiated from the baseline. IR(cm^{-1}): 3062, 3020, 2964, 1644, 1599, 1511, 1460, 1276, 1081, 1059, 976, 755, 698, 571. MS [M+H]$^{+}$: found: 307.1485, calculated: 307.1487.

Literature values for comparison:[1] 1,2,4-Triphenylbenzene ^{1}H NMR (300 MHz, $CDCl_3$): δ 7.15-7.22 (m, 10H), 7.37-7.52 (m, 4H), 7.64-7.69 (m, 4H)

1,3,5-Triphenylbenzene ^{1}H NMR (300 MHz, $CDCl_3$): δ 7.36-7.41 (m, 3H), 7.48 (t, J = 7.6 Hz, 6H), 7.70 (d, J = 7.9 Hz, 6H), 7.79 (s, 3H)

*Trimerisation of t-butylacetylene using dimer **1a** (cation **2a**)*: Dimer **1a** (28.38 mg, 0.105 mmol) and $[Ph_3C][B(C_6F_5)_4]$ (96.85 mg, 0.105 mmol) was dissolved in 2 mL of THF (catalytic loading of 10 mol%). *t*-butylacetylene was added (1.05 mmol, 0.13 mL) and the reaction solution was stirred at 30 °C for 24 h. The reaction was quenched with an excess of MeOH and the resultant light brown solution was removed *via* cannula, leaving behind a brownish-white solid, which was washed with MeOH a few times and dried *in vacuo*. Yield 59.6 mg, 69%. TON: 7. IR (cm^{-1}): 2963, 1644, 1612, 1513, 1461, 1373, 1363, 1259, 1019. MS $[M+H]^+$: found: 247.2429, calculated: 247.2426.

*Trimerisation of t-butylacetylene using dimer **1f** (cation **2f**)*: Dimer **1f** (52.36 mg, 0.105 mmol) and $[Ph_3C][B(C_6F_5)_4]$ (96.85 mg, 0.105 mmol) was dissolved in 2 mL of THF (catalytic loading of 10 mol%). *t*-butylacetylene was added (1.05 mmol, 0.13 mL) and the reaction solution was stirred at 30 °C for 24 h. The reaction was quenched with an excess of MeOH and the resultant light brown solution was removed *via* cannula, leaving behind a brownish-white solid, which was washed with MeOH a few times and dried *in vacuo*. Yield 52.4 mg, 61%. TON: 6. IR (cm^{-1}): 2960, 1643, 1612, 1590, 1514, 1462, 1374, 1366, 1258, 1021. MS $[M+H]^+$: found: 247.2429, calculated: 247.2426.

*Polymerisation of 1-hexene using dimer **1a** (cation **2a**)*: To a solution of dimer **1a** (28.38 mg, 0.105 mmol) and $[Ph_3C][B(C_6F_5)_4]$ (96.85 mg, 0.105 mmol) in toluene (3 mL) (10 mol% catalytic loading), 1-hexene was added (1.05 mmol, 0.13 mL). The reaction was stirred for 30 min at room temperature. Quenching with an excess of methanol turned the orange solution clear. The solvents were taken off *in vacuo* and the product (white solid) was washed with MeOH. Yield 105.2 mg. ^1H NMR (500 MHz, 25 °C, d-chloroform): δ 1.18–1.36 (mult, bd, H(1) & H(3)–H(5)), 0.91 (mult, bd, H2), 0.81–0.90 (mult, bd, H6) ppm. ^{13}C NMR (126 MHz, 25 °C, d-chloroform): δ 37.6 (C(1)), 34.2 (C(3)), 30.41 (C(2)), 29.9 (C(4)), 22.8 (C(5)), 13.9 (C(6)) ppm. GPC: M_n = 4641 g mol^{-1}, M_w = 4899 g mol^{-1}, $Đ$ = 1.1. IR (cm^{-1}): 2957, 2954, 2854, 1644, 1513, 1456, 1375, 1276, 1081, 974, 755, 661.

*Polymerisation of 1-hexene using dimer **1f** (cation **2f**)*: To a solution of dimer **1f** (52.36 mg, 0.105 mmol) and $[Ph_3C][B(C_6F_5)_4]$ (96.85 mg, 0.105 mmol) in toluene (3 mL) (10 mol%

catalytic loading), 1-hexene was added (1.05 mmol, 0.13 mL). The reaction was stirred for 30 min at room temperature. Quenching with an excess of methanol turned the orange solution clear. The solvents were taken off *in vacuo* and the product (white solid) was washed with MeOH. Yield 286.4 mg. ^{1}H NMR (500 MHz, 25 °C, d-chloroform): δ 1.36–1.48 (mult, bd, H(1) & H(3)–H(5)), 1.04 (mult, bd, H2), 0.92–1.03 (mult, bd, H6). ^{13}C NMR (126 MHz, 25 °C, d-chloroform): δ 39.9 (C(1)), 34.5 (C(3)), 30.1 (C(2)), 29.8 (C(4)), 22.9 (C(5)), 14.1 (C(6)). GPC: M_n = 4518 g mol^{-1}, M_w = 4761 g mol^{-1}, $Đ$ = 1.1. IR (cm^{-1}): 2957, 2954, 2859, 1644, 1513, 1456, 1374, 1272, 1081, 973, 755, 661.

*Polymerisation of cyclohexene using dimer **1a** (cation **2a**)*: To a solution of dimer **1a** (28.38 mg, 0.105 mmol) and $[Ph_3C][B(C_6F_5)_4]$ (96.85 mg, 0.105 mmol) in toluene (3 mL) (10 mol% catalytic loading), cyclohexene was added (1.05 mmol, 0.11 mL). The reaction was stirred for 24 h at 80 °C. Quenching with an excess of methanol turned the brown solution clear. The solvents were taken off *in vacuo* and the product (brownish-white solid) was washed with MeOH. Yield 147.6 mg. ^{1}H NMR (500 MHz, 25 °C, d-chloroform): δ 1.41–2.53 (mult, CH_2 groups in cyclohexane) ppm. ^{13}C NMR (126 MHz, 25 °C, d-chloroform): δ 19.0, 19.4, 19.5, 21.0, 21.5, 21.6, 26.3 (4 signals), 26.4, 26.5, 27.0, 27.1, 27.3 (3 signals), 30.6, 33.7, 33.8, 33.9, 34.6, 34.6, 34.7, 34.7 ppm. GPC: M_n = 4883 g mol^{-1}, M_w = 5078 g mol^{-1}, $Đ$ = 1.0. IR (cm^{-1}): 2924, 2850, 1643, 1460, 907, 896.

*Polymerisation of cyclohexene using dimer **1f** (cation **2f**)*: To a solution of dimer **1f** (52.36 mg, 0.105 mmol) and $[Ph_3C][B(C_6F_5)_4]$ (96.85 mg, 0.105 mmol) in toluene (3 mL) (10 mol% catalytic loading), cyclohexene was added (1.05 mmol, 0.11 mL). The reaction was stirred for 24 h at 80 °C. Quenching with an excess of methanol turned the brown solution clear. The solvents were taken off *in vacuo* and the product (brownish-white solid) was washed with MeOH. Yield 263.3 mg. ^{1}H NMR (500 MHz, 25 °C, d-chloroform): δ 1.40–2.50 (mult, CH_2 groups in cyclohexane) ppm. ^{13}C NMR (126 MHz, 25 °C, d-chloroform): δ 18.9, 19.3, 19.4, 21.0, 21.5, 21.5, 26.2, 26.2, 26.3, 26.4, 26.4, 27.0, 27.1, 27.2, 27.3, 27.4, 31.4, 33.7, 33.8, 33.8, 34.6, 34.6, 34.6, 34.7 ppm. GPC: M_n = 5100 g mol^{-1}, M_w = 5380 g mol^{-1}, $Đ$ = 1.0. IR (cm^{-1}): 2923, 2850, 1644, 1458, 906, 890.

Additional NMR spectra

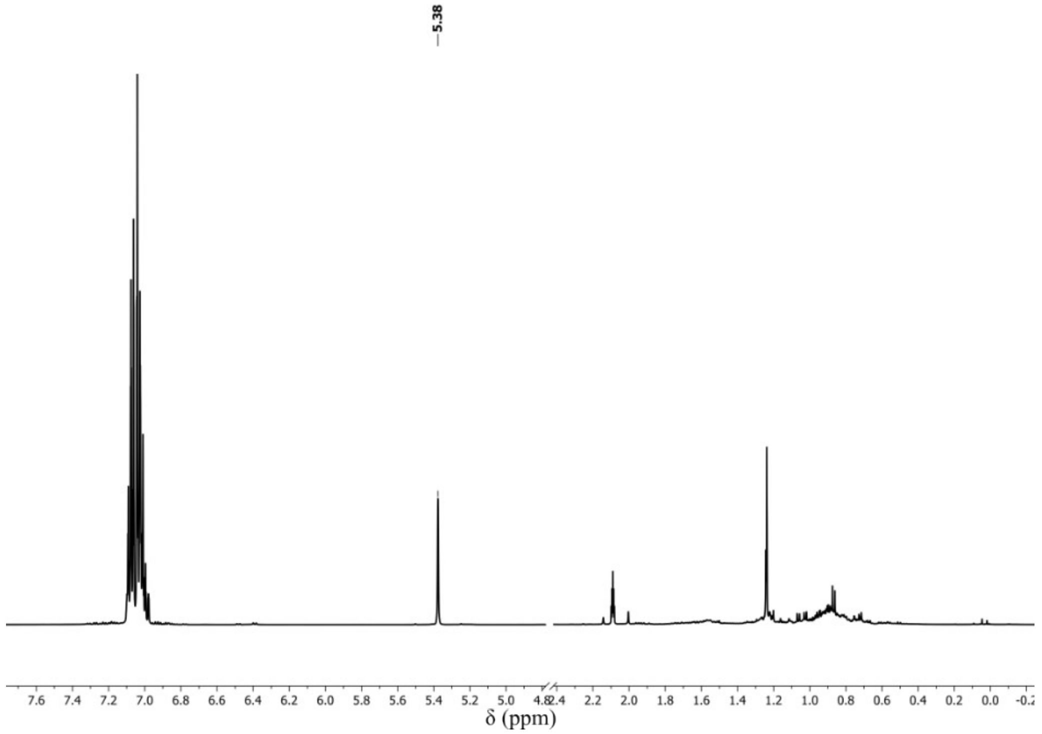

Figure S3. ^{1}H NMR (25 °C, d$_8$-toluene, 500.1 MHz) spectrum of the reaction of **1e** with $[Ph_3C][B(C_6F_5)_4]$ (1:1). The extraction of the ^{i}Bu group is expected to result in the formation Ph_3CH and release of isobutene. The peak at *δ* 5.38 ppm represents Ph_3CH.

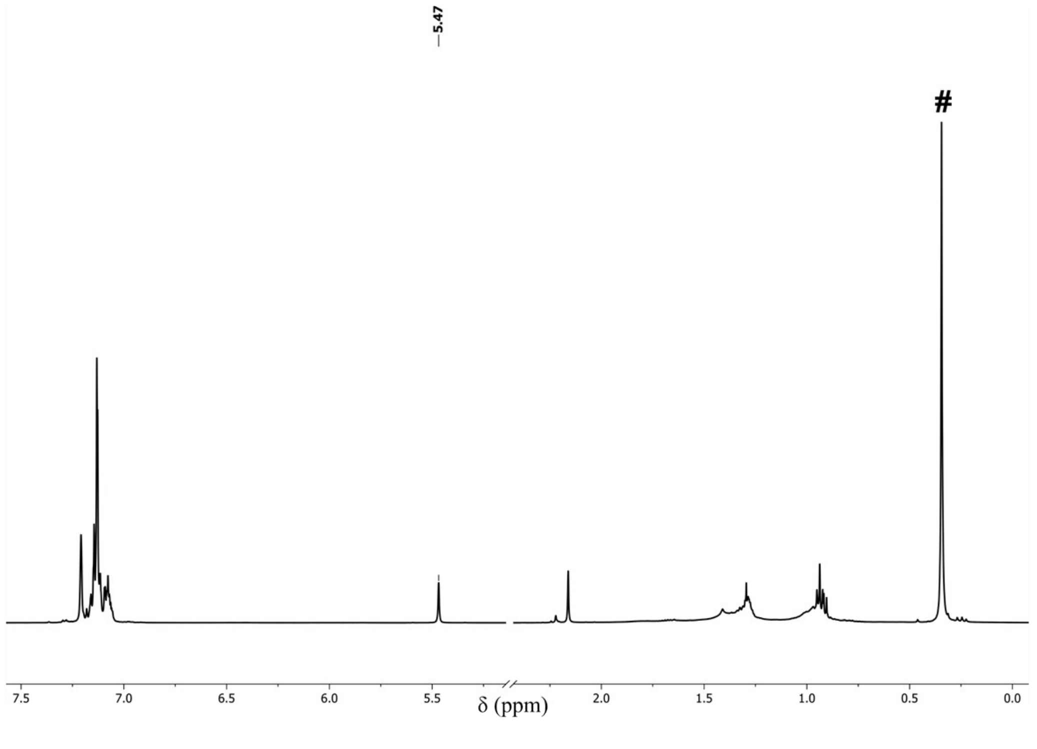

Figure S4. ^{1}H NMR (25 °C, d$_6$-benzene, 500.1 MHz) spectrum of the reaction of **1f** with $[Ph_3C][B(C_6F_5)_4]$ (1:1). The extraction of the ^{i}Bu group is expected to result in the formation Ph_3CH and release of isobutene. The peak at *δ* 5.47 ppm represents Ph_3CH. # vacuum grease.

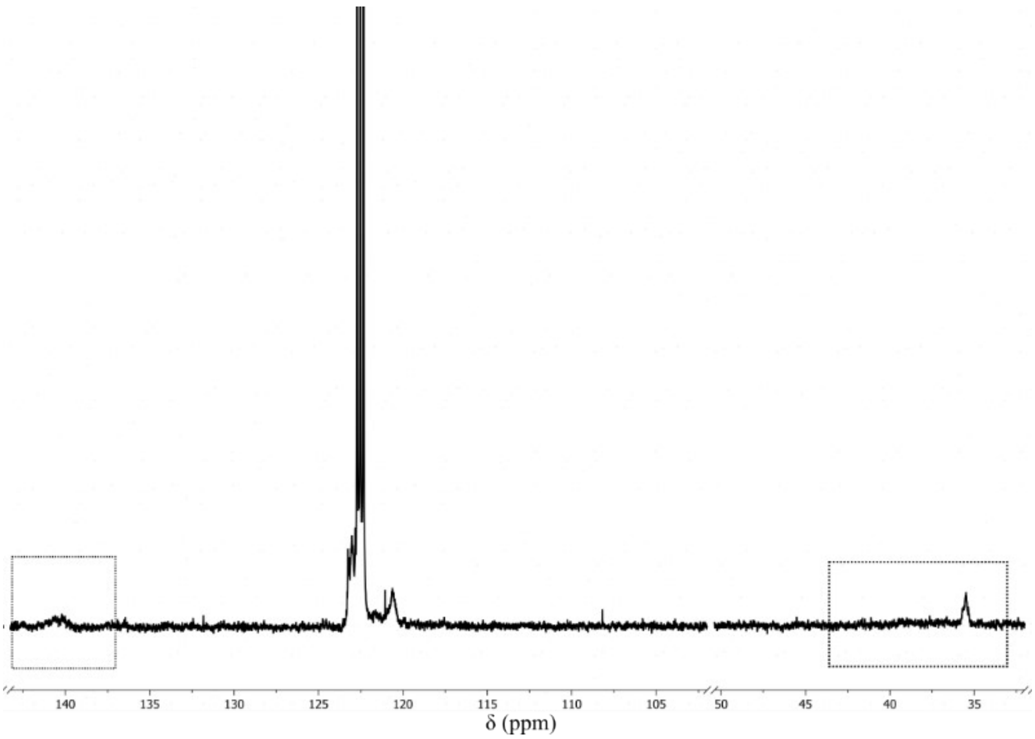

Figure S5. ^{13}C NMR (25 °C, d_8-toluene, 126 MHz) spectrum of atactic polystyrene prepared using a 10 mol% loading of only $[Ph_3C][B(C_6F_5)_4]$ at room temperature (control experiment without any aluminium pyridiyl precatalyst.

IR spectra

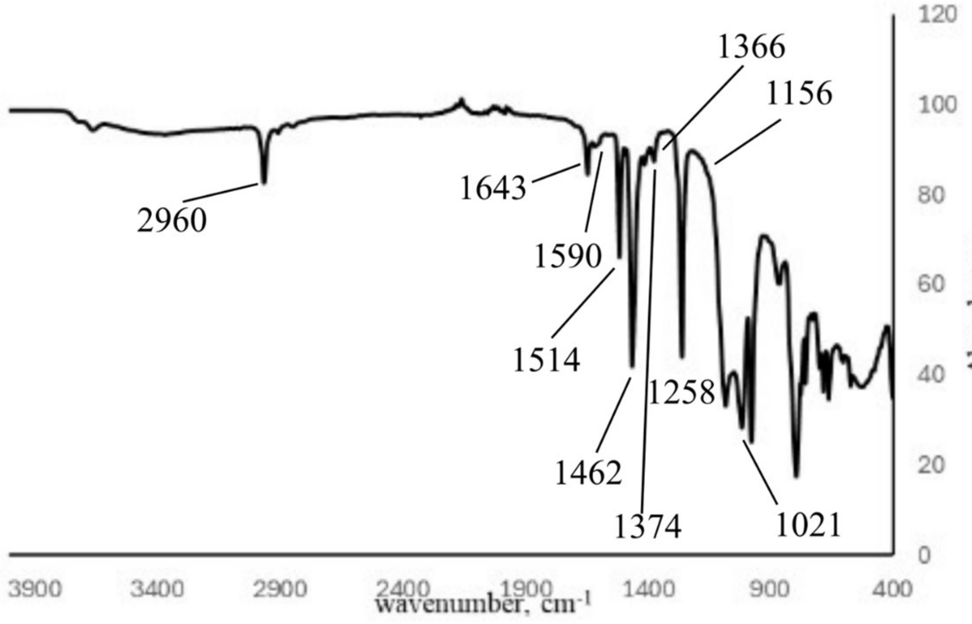

Figure S6. IR spectrum of the trimerisation product of *t*-butylacetylene (1,3,6-tri-*t*-butylfulvene) prepared using a 10 mol% catalytic loading of precatalyst **1f** and $[Ph_3C][B(C_6F_5)_4]$ (cation **2f**).

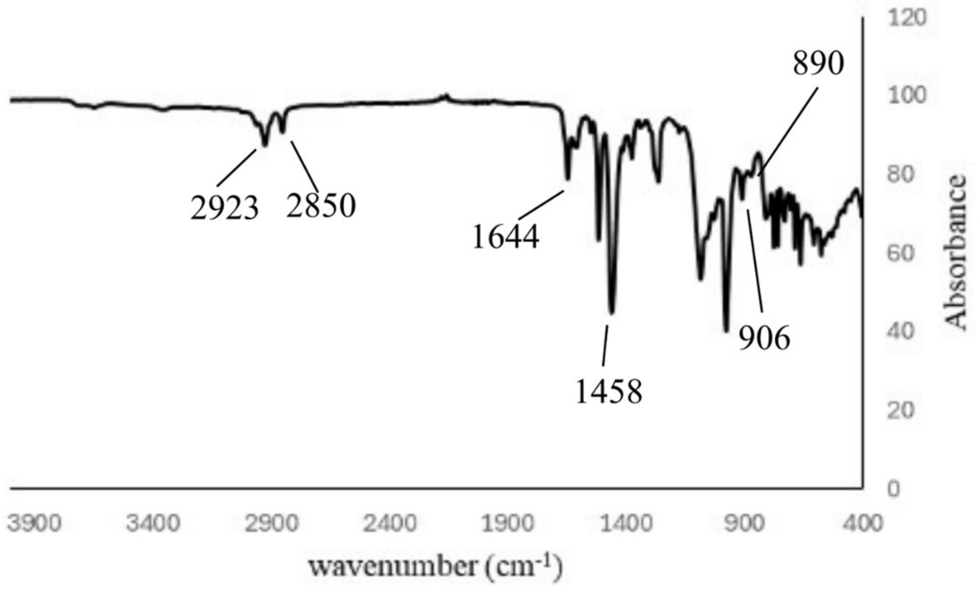

Figure S7. IR spectrum of the cyclohexene polymerisation product prepared using a 10 mol% catalytic loading of precatalyst **1f** and $[Ph_3C][B(C_6F_5)_4]$ (cation **2f**).

SEC traces

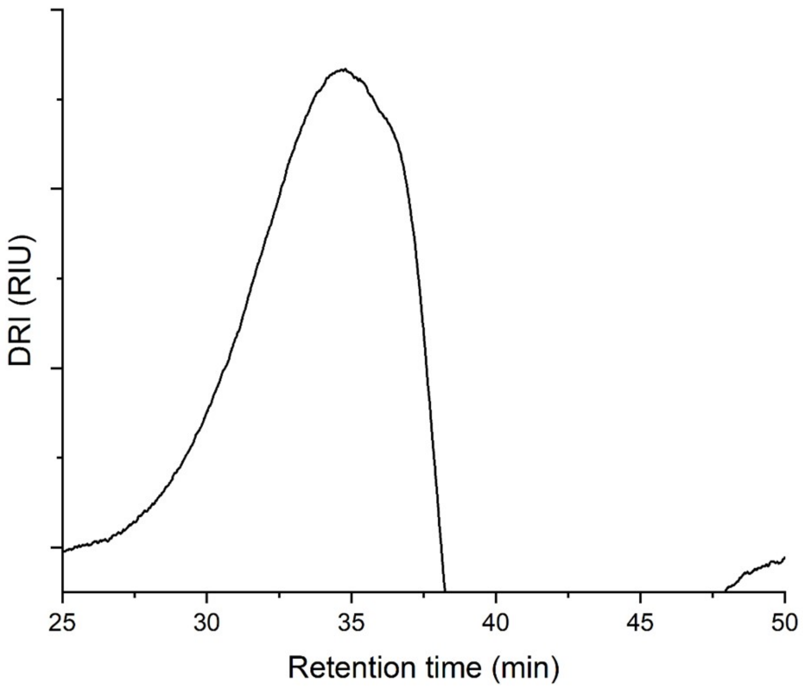

Figure S8. GPC trace of styrene polymerisation product using a 0.5 mol% catalytic loading of cation **2a** at room temperature. The peak at ~36 min is a known solvent peak.

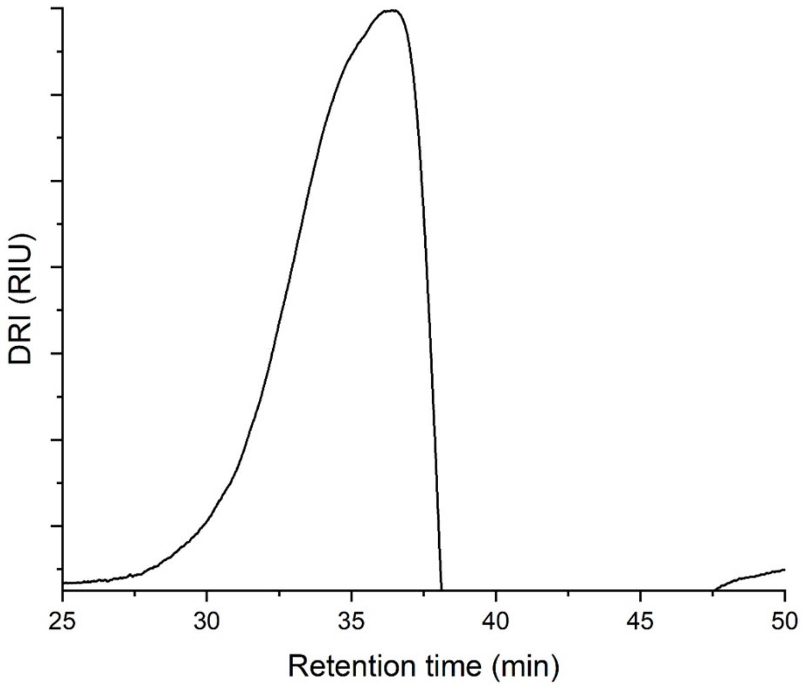

Figure S9. GPC trace of styrene polymerisation product using a 2.5 mol% catalytic loading of cation **2a** at room temperature. The peak at ~36 min is a known solvent peak.

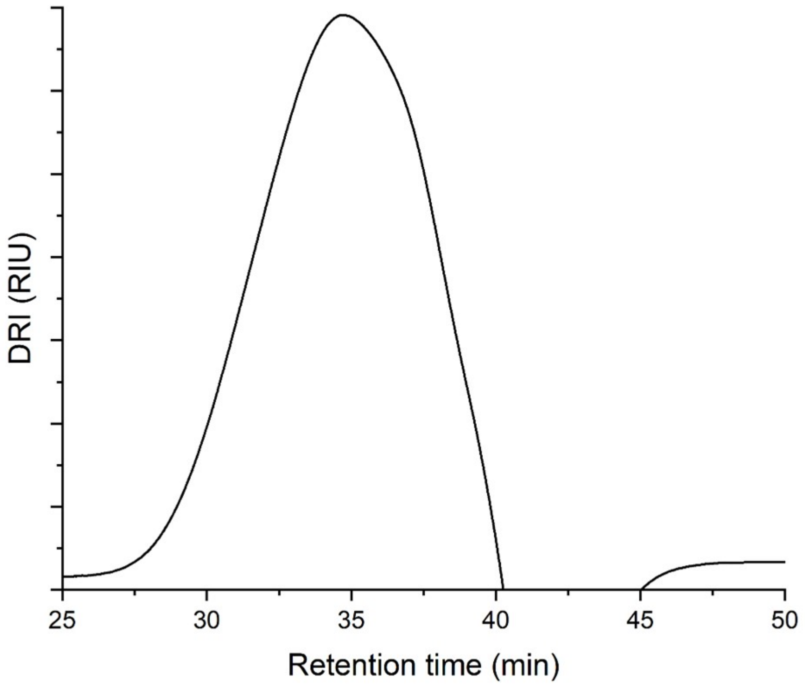

Figure S10. GPC trace of styrene polymerisation product using a 0.5 mol% catalytic loading of cation **2a** at −15 °C. The peak at ~36 min is a known solvent peak.

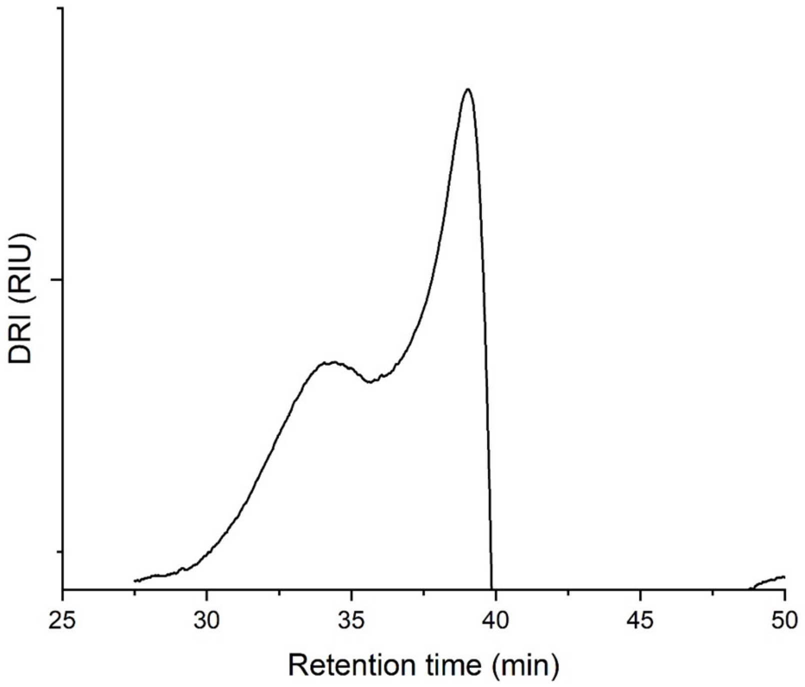

Figure S11. GPC trace of styrene polymerisation product using a 2.5 mol% catalytic loading of cation **2a** at −15 °C. The peak at ~36 min is a known solvent peak.

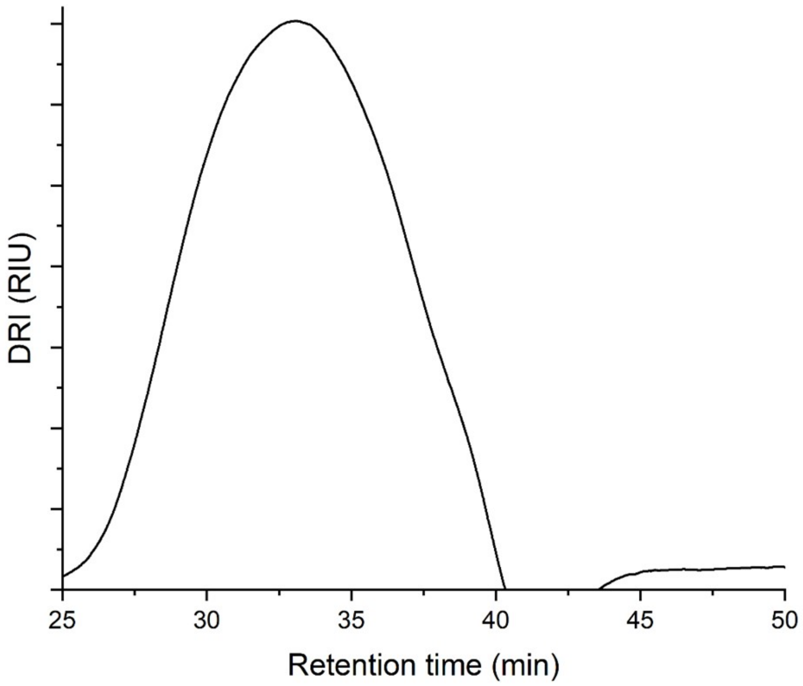

Figure S12. GPC trace of styrene polymerisation product using a 0.5 mol% catalytic loading of cation **2b** at 0 °C. The peak at ~36 min is a known solvent peak.

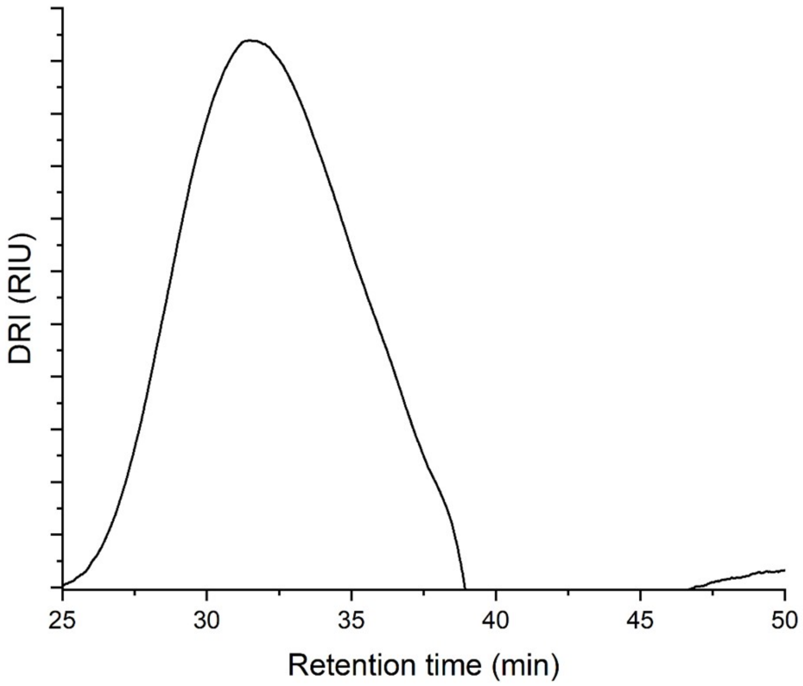

Figure S13. GPC trace of styrene polymerisation product using a 0.5 mol% catalytic loading of cation **2c** at 0 °C. The peak at ~36 min is a known solvent peak.

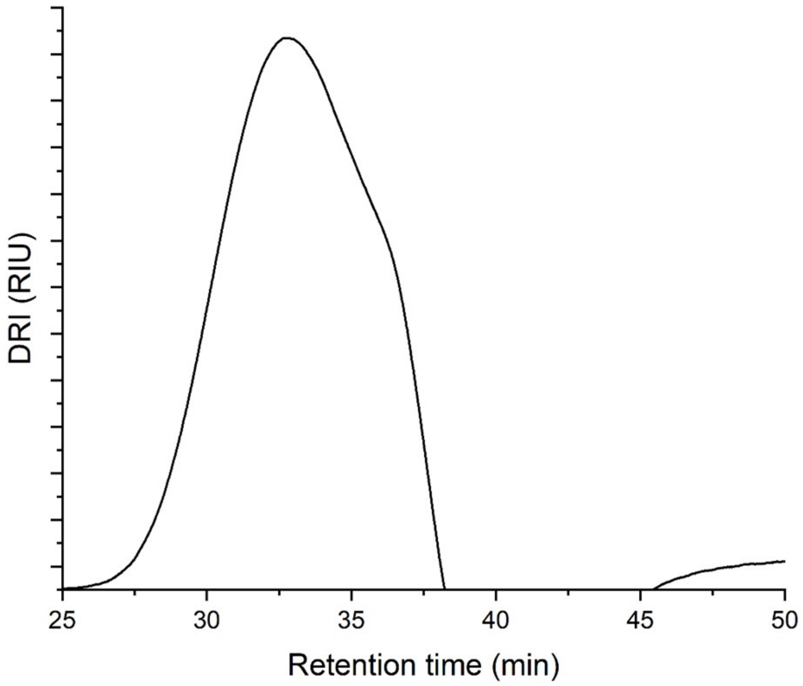

Figure S14. GPC trace of styrene polymerisation product using a 0.5 mol% catalytic loading of cation **2e** at −15 °C. The peak at ~36 min is a known solvent peak.

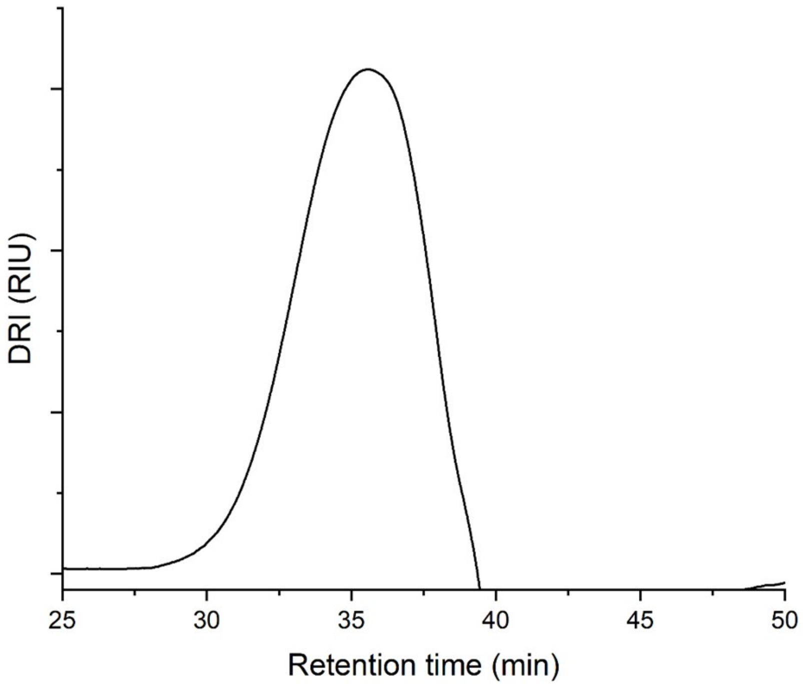

Figure S15. GPC trace of styrene polymerisation product using a 2.5 mol% catalytic loading of cation **2e** at −15 °C. The peak at ~36 min is a known solvent peak.

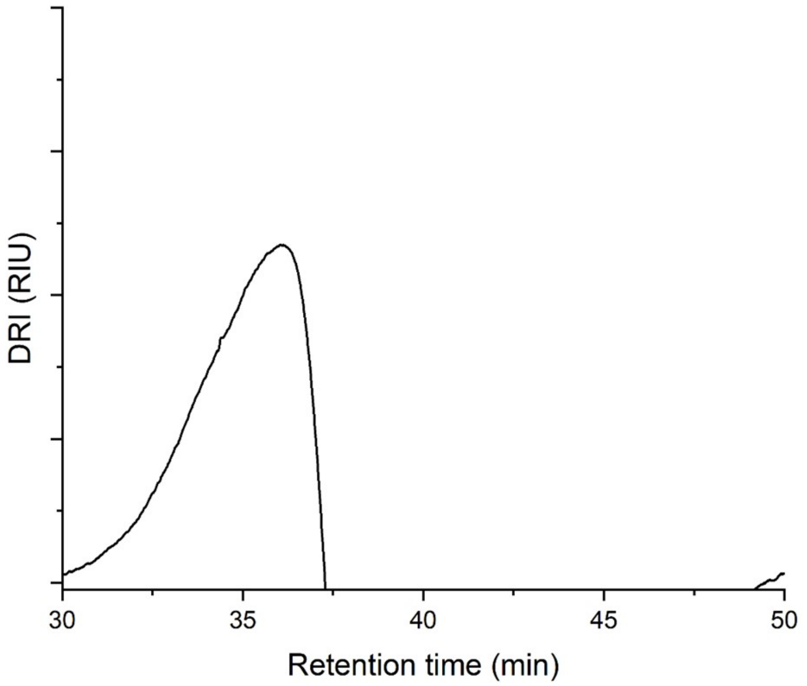

Figure S16. GPC trace of styrene polymerisation product using a 5 mol% catalytic loading of cation **2e** at −15 °C. The peak at ~36 min is a known solvent peak.

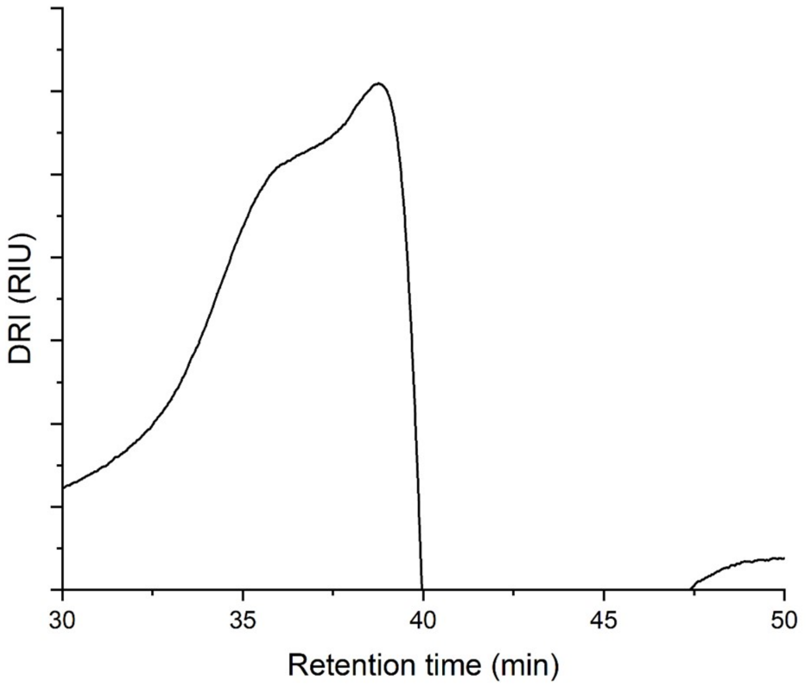

Figure S17. GPC trace of styrene polymerisation product using a 10 mol% catalytic loading of cation **2e** at −15 °C. The peak at ~36 min is a known solvent peak.

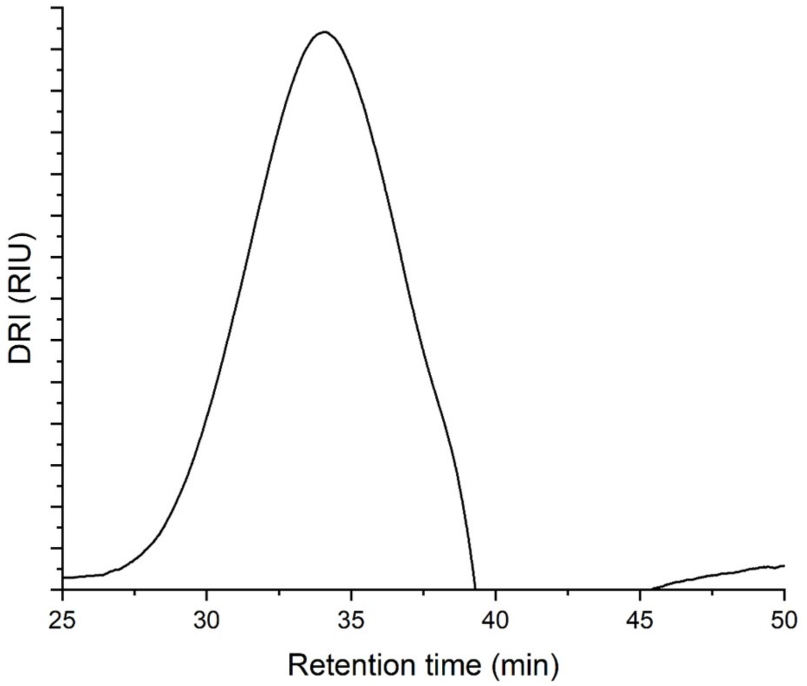

Figure S18. GPC trace of styrene polymerisation product using a 0.5 mol% catalytic loading of cation **2f** at 0 °C. The peak at ~36 min is a known solvent peak.

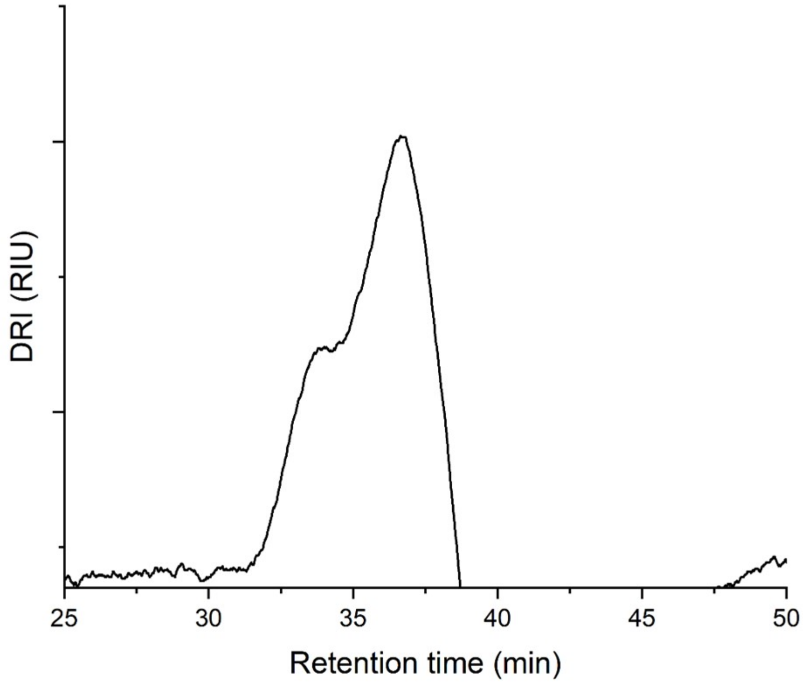

Figure S19. GPC trace of 1-hexene polymerisation product using a 10 mol% catalytic loading of cation **2a**. The peak at ~36 min is a known solvent peak.

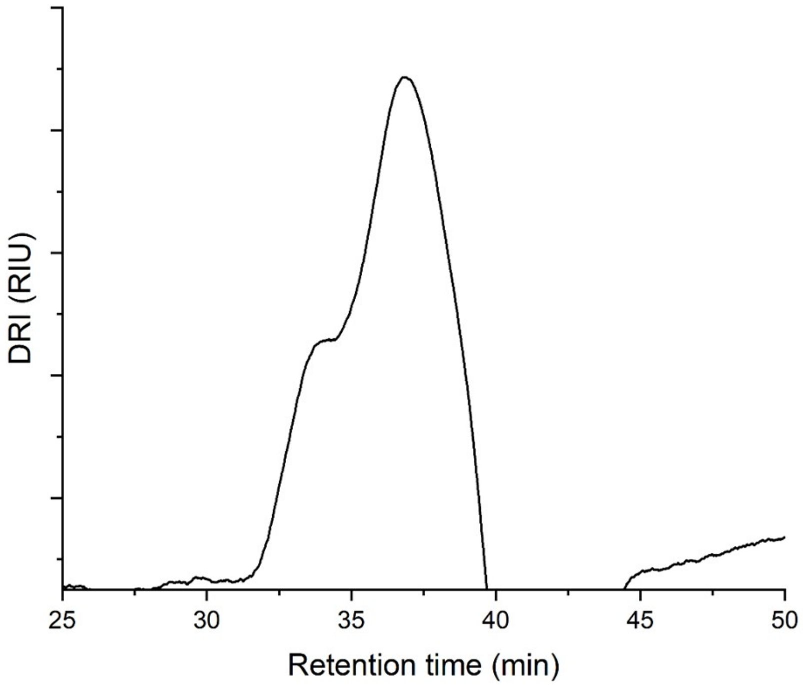

Figure S20. GPC trace of 1-hexene polymerisation product using a 10 mol% catalytic loading of cation **2f**. The peak at ~36 min is a known solvent peak.

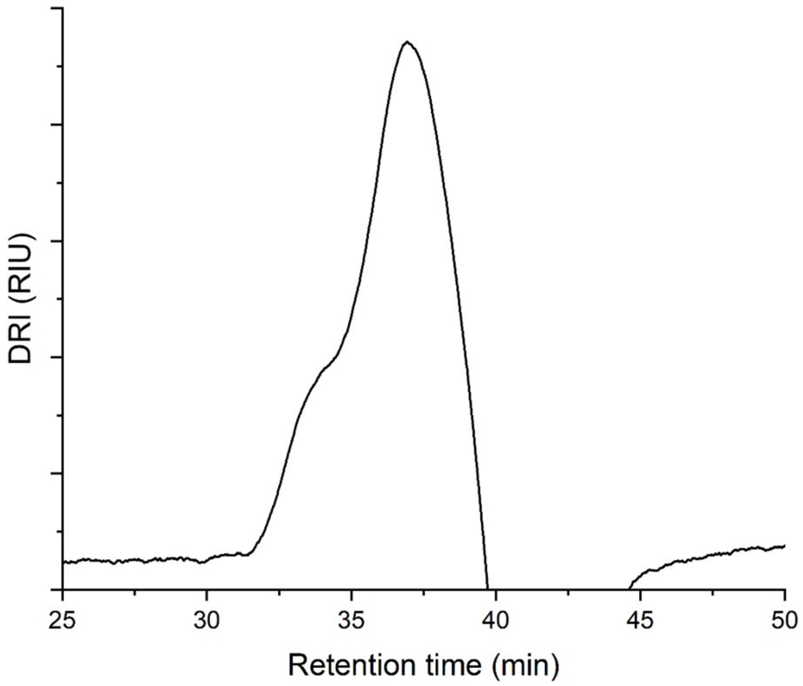

Figure S21. GPC trace of cyclohexene polymerisation product using a 10 mol% catalytic loading of cation **2a**. The peak at ~36 min is a known solvent peak.

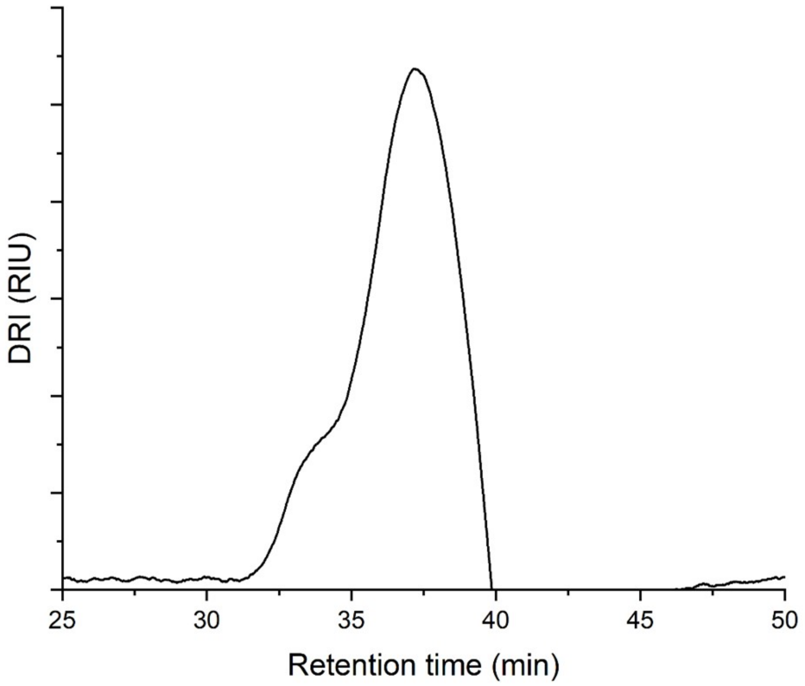

Figure S22. GPC trace of cyclohexene polymerisation product using a 10 mol% catalytic loading of cation **2f**. The peak at ~36 min is a known solvent peak.

DSC thermal analysis

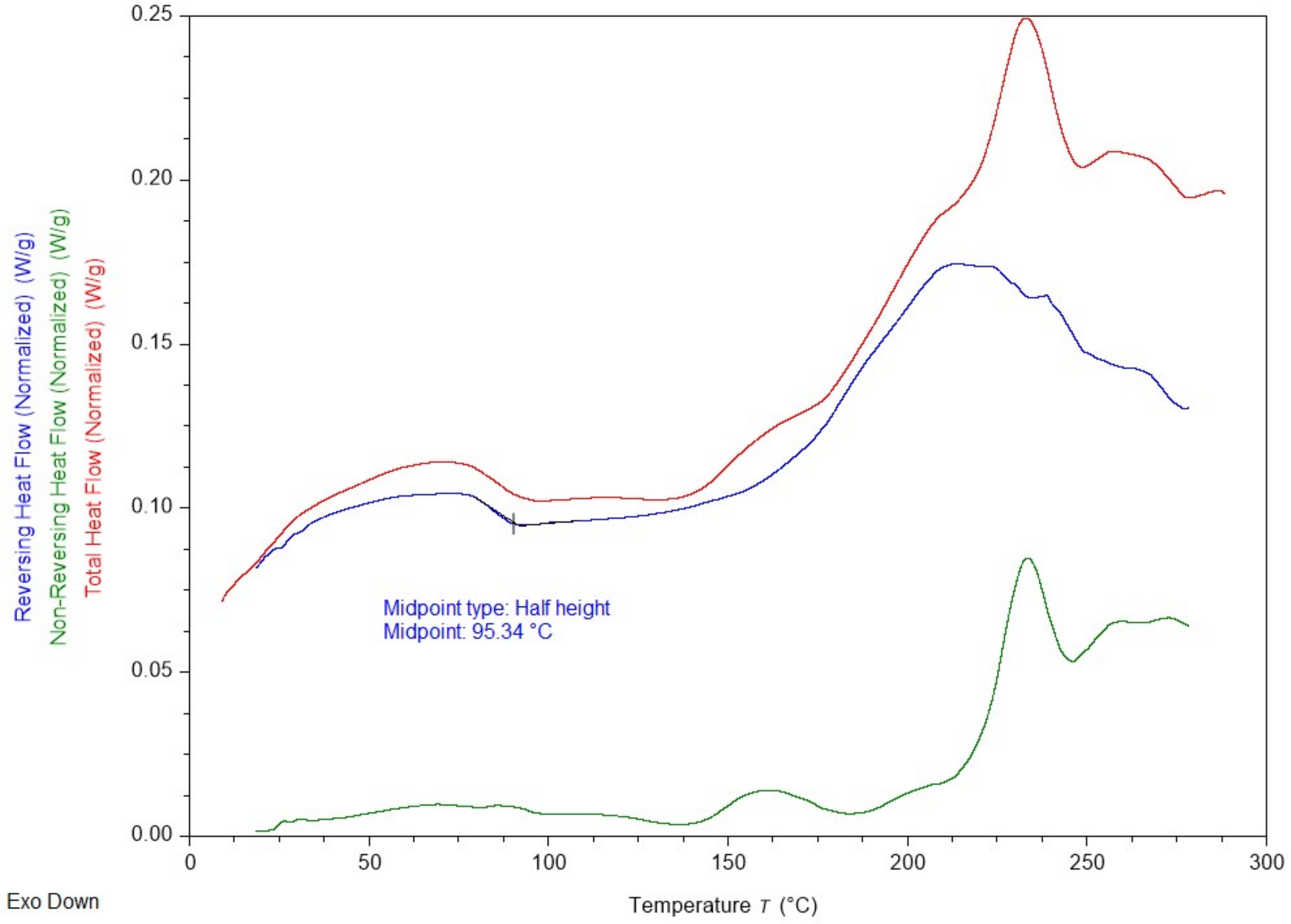

Figure S23. DSC curve of (atactic) styrene polymerisation product using a 0.5 mol% catalytic loading of cation **2b** at 0 °C. T_g= 95.34 °C.

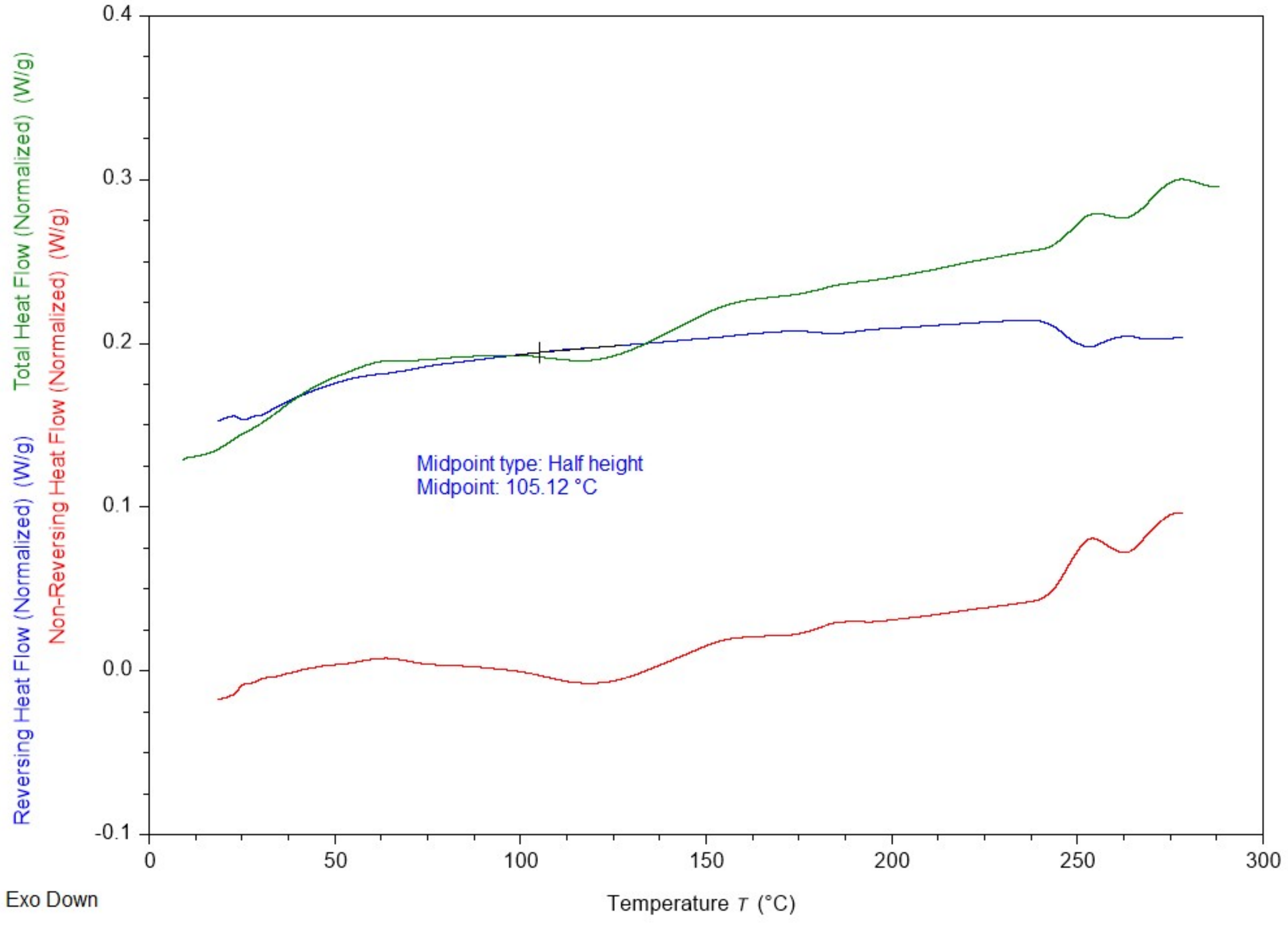

Figure S24. DSC curve of (atactic+syndiotatctic) styrene polymerisation product using a 0.5 mol% catalytic loading of cation **2f** at 0 °C. T_g= 105.12 °C.

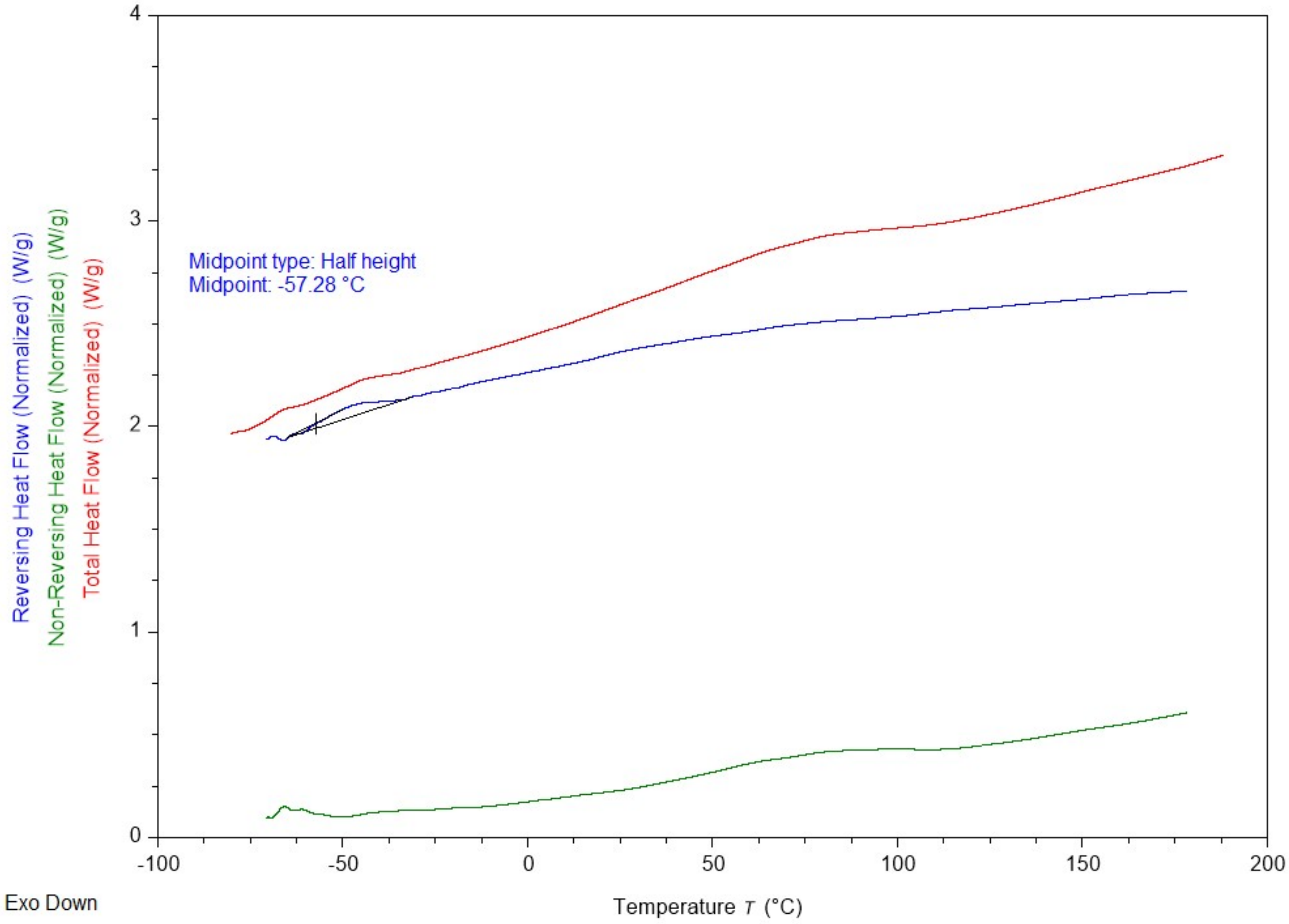

Figure S25. DSC curve of 1-hexene polymerisation product using a 10 mol% catalytic loading of cation **2a**. T_g= − 57.28 °C.

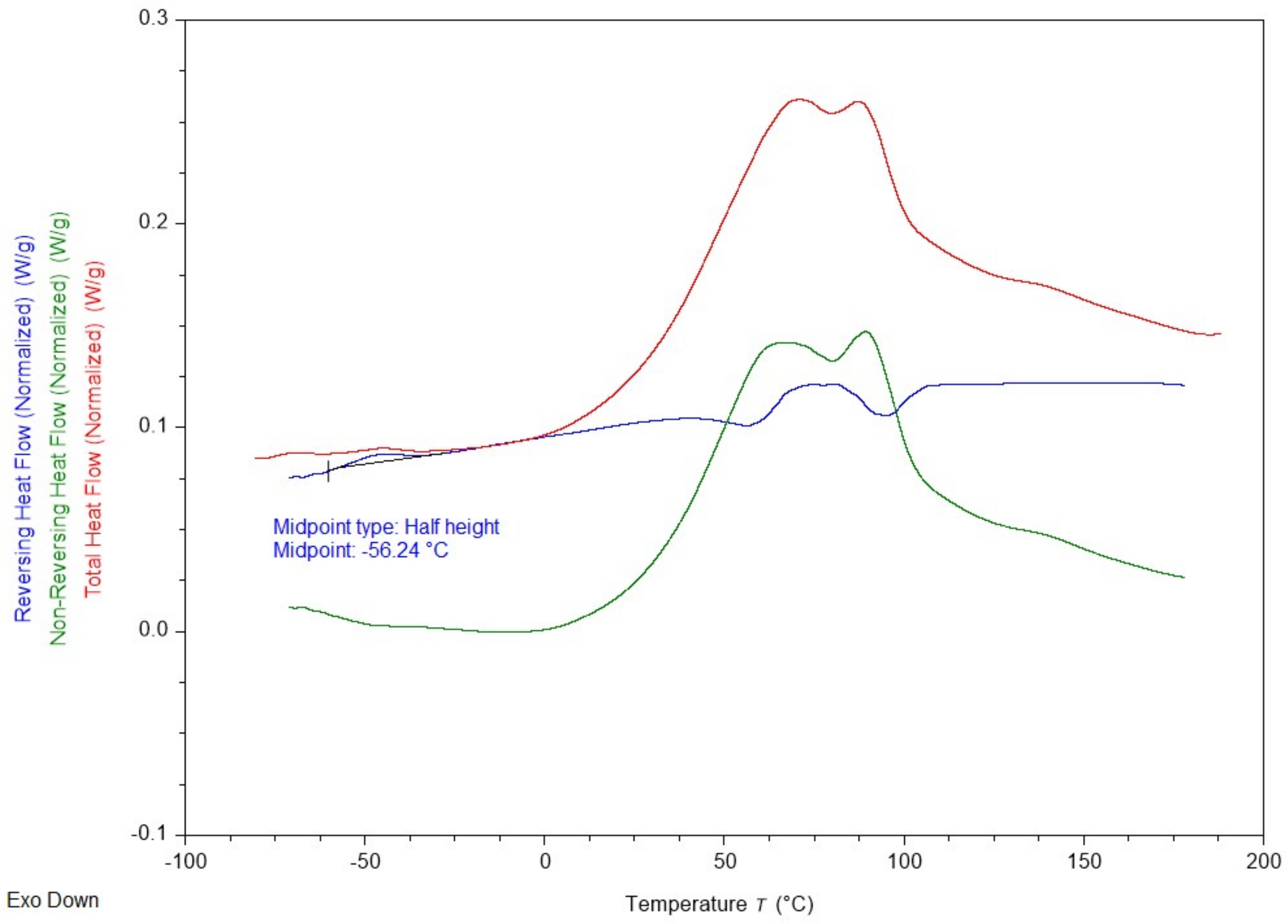

Figure S26. DSC curve of 1-hexene polymerisation product using a 10 mol% catalytic loading of cation **2f**. T_g= − 56.24 °C.

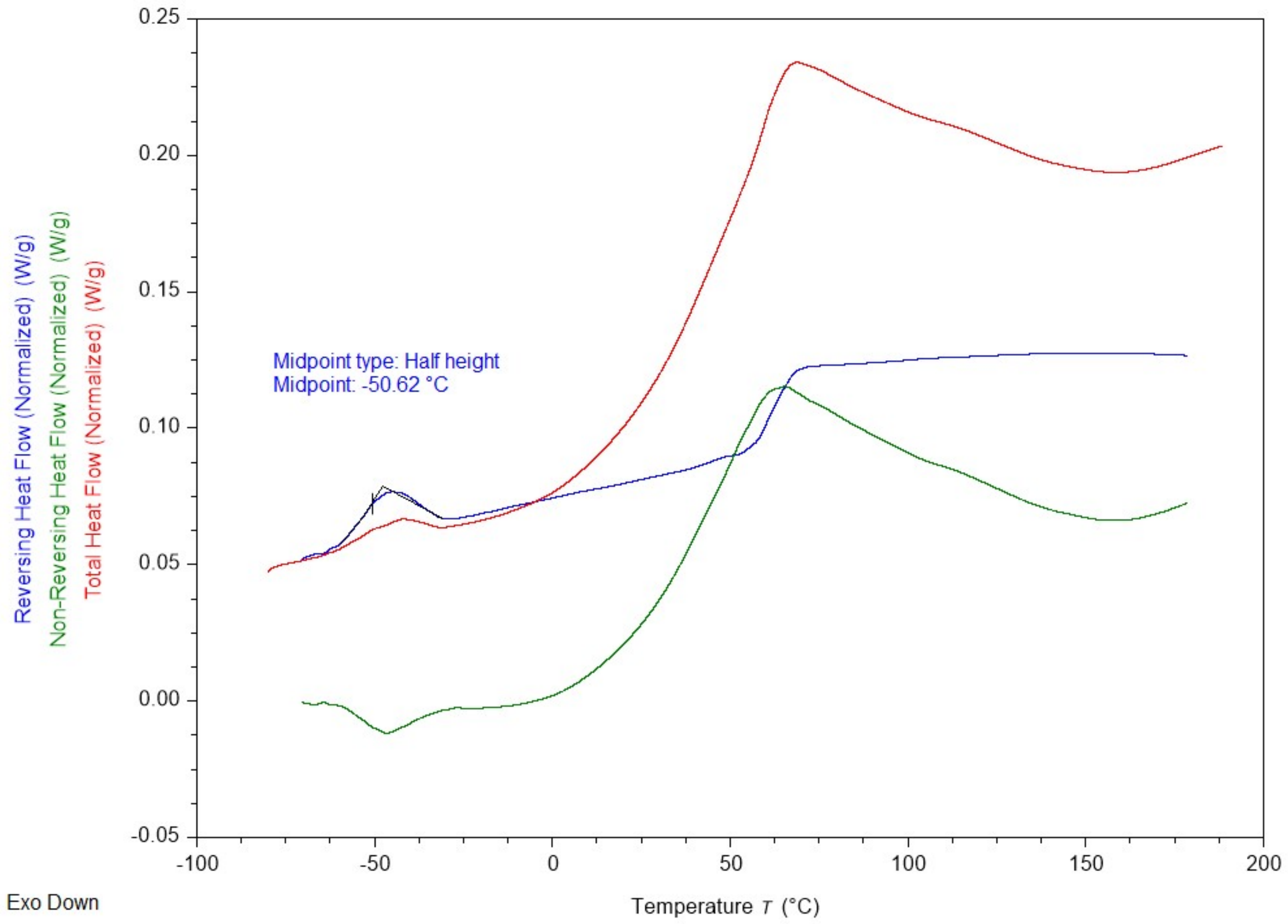

Figure S27. DSC curve of cyclohexene polymerisation product using a 10 mol% catalytic loading of cation **2a**. $T_g = -50.62$ °C.

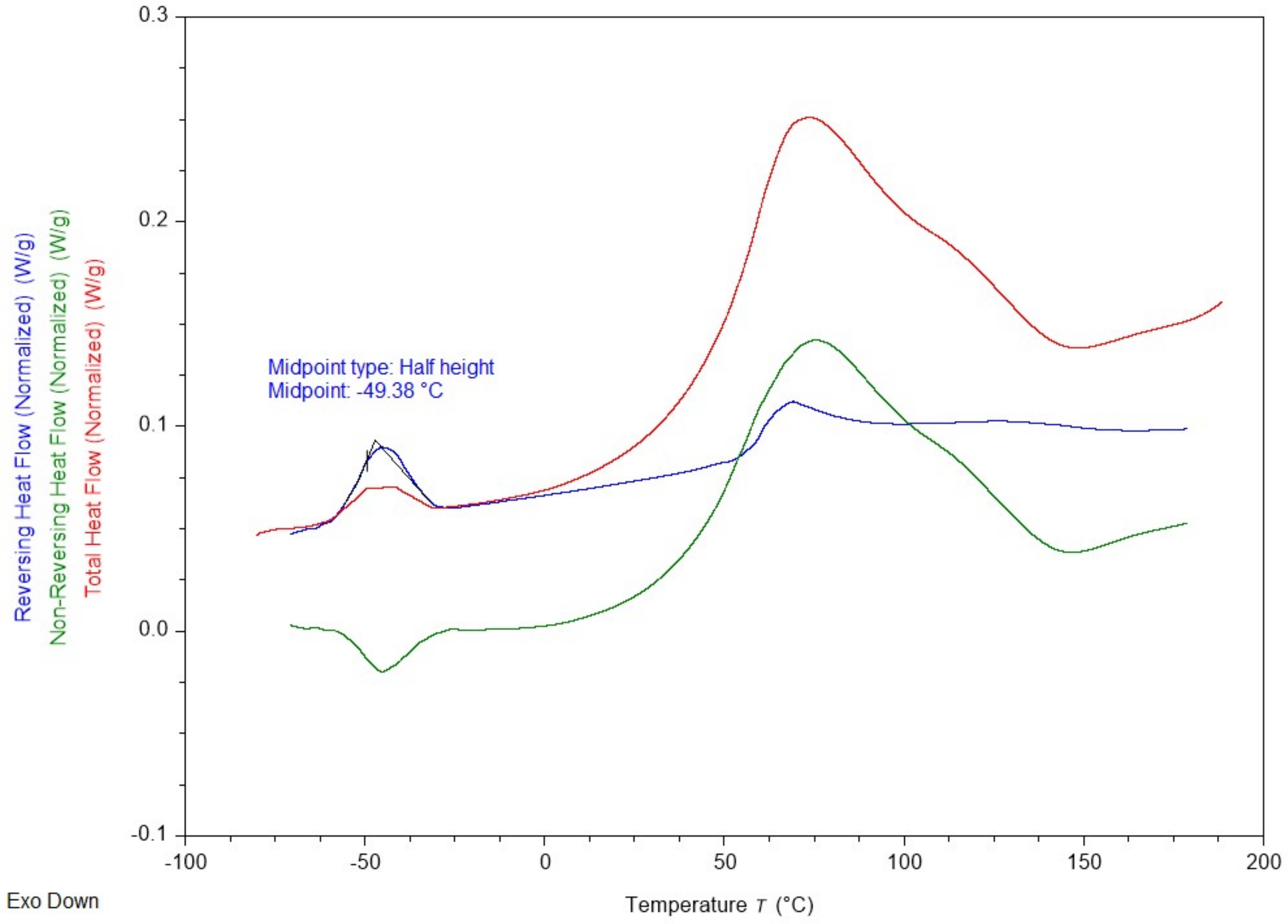

Figure S28. DSC curve of cyclohexene polymerisation product using a 10 mol% catalytic loading of cation **2f**. $T_g = -49.28$ °C.

Computational methods

Mechanistic studies

Density Functional Theory (DFT) calculations were carried out using Gaussian 16 (Revision B.01).[2] Structural optimisations were performed at the B3LYP-D3/6-31G(d) level of theory.[3–6] Frequency analyses were conducted to validate the optimised structures, ensuring they corresponded to either energy minima or first-order saddle points on the potential surface. Single-point energy values of optimised structures were computed at the ωB97X-D/6-311++G(d,p)[7] level of theory. In order to make the structure compatible with the forcefield parameterisation, Al was replaced with Si to produce a pseudo structure for conformational searching. Conformational searches were conducted in MacroModel (v11.7) with Maestro (release 2024-03)[8] using the OPLS4 force field with the mixed torsional / low-mode sampling method. A setting of 1000 as the maximum number of steps and the maximum number of steps per rotatable bond was used. Conformers within an energy window of 5.0 kcal mol^{-1} were saved for further analyses. CYLView 2 was used to generate images of structures.[9]

Molecular surface analyses

DFT calculations were carried out using Orca *v.* 6.0.1.[10] Single-point energy values of structures optimised as above were computed at the ωB97X-D3(BJ)/def2-TZVP level of theory (with the auxiliary basis set def2/J).[11-13] Molecular electrostatic potentials (ESP) were calculated using the `orca_vpot` functionality of Orca 6.0.1, whereas average local ionisation energy (ALIE) and local electron attachment energy (LEAE) calculations were carried out using Multiwfn *v.* 3.8;[10,14,15] in all cases, the properties were computed on the wavefunctions from the aforementioned single-point calculations (ωB97X-D3(BJ)/def2-TZVP). All three properties were mapped on the surfaces of the ρ = 0.001 a.u. isodensity envelopes and were plotted with ChimeraX *v.* 1.10.[16]

Topographic steric analyses

Topographic steric maps were generated with SambVca *v.* 2.1, including H-atoms; the parameter values used for the generation of the isocontours and calculation of buried volumes were those recommended by the original authors of the program (Bondi radii scaled by 1.17, sphere radius R = 3.5 Å, distance from Al centre d = 0.0 Å; a slightly finer mesh spacing of s = 0.08 Å was used over the default).

Fluoride ion affinities (FIA)

	**G**	**ΔG**	**FIA**
CF_2O	-313.03413		0 (by definition)
CF_3O^-	-412.97138	–99.937253	
**[Int1]**$^+$	-1099.6194		–0.193817
**Int1**-F	-1199.7505	–100.13107	
Ph_3C^+	-732.48536		–0.163814
Ph_3CF	-832.58643	–100.10107	
$AlCl_3$	-1623.28800		–0.107878
$[AlCl_3F]^-$	-1723.33320	–100.04513	

Calculated at ωB97X-D/6-311++G(d,p)//B3LYP-D3/6-31G(d)

Qualitative molecular surface analysis

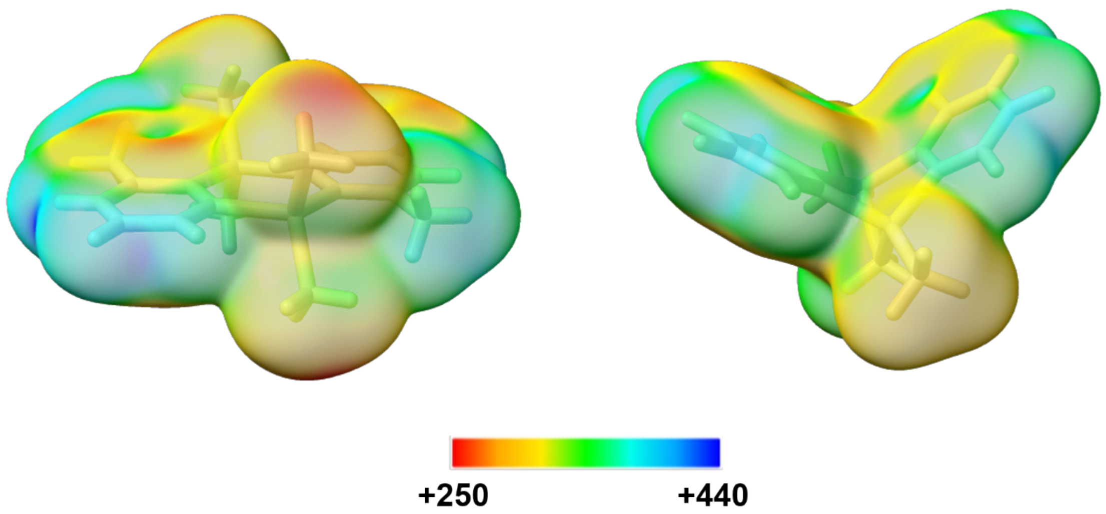

Figure S29. Average local ionisation energy (ALIE) maps for one of the neutral pre-catalysts (**1b**, right) and a catalytically active cationic complex (**2a**, $[MeAl(2\text{-py})_2(\mu\text{-Me})AlMe]^+$ as per our calculations, left) mapped onto the 0.001 a.u. isodensity envelope; the ALIE scale is in kcal mol^{-1}. The global minimum value is located clearly on the terminal methyl of the pre-catalyst, indicating the high electrophilicity thereof.

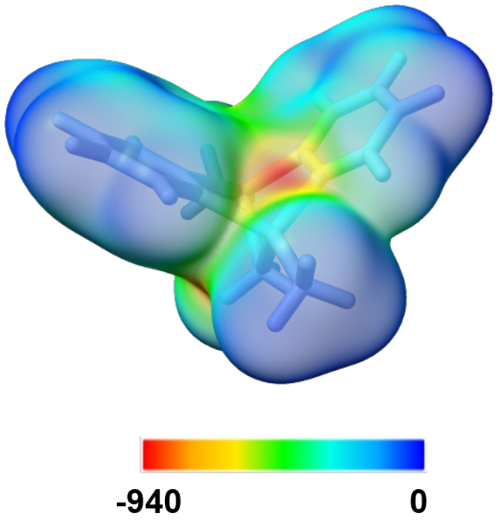

Figure S30. Local electron attachment energy (LEAE) map for a catalytically active cationic complex (**2a**, $[MeAl(2\text{-py})_2(\mu\text{-Me})AlMe^+]$) mapped onto the 0.001 a.u. isodensity envelope; the LEAE scale is in kcal mol^{-1}. Note that a corresponding calculation is not possible for the neutral pre-catalysts, as in their case all unoccupied orbitals have positive energies. The LEAE maximum value, highly concentrated on the locus of the Al σ-hole, demonstrates the high nucleophilicity thereof.

Stereochemistry of chain transfer

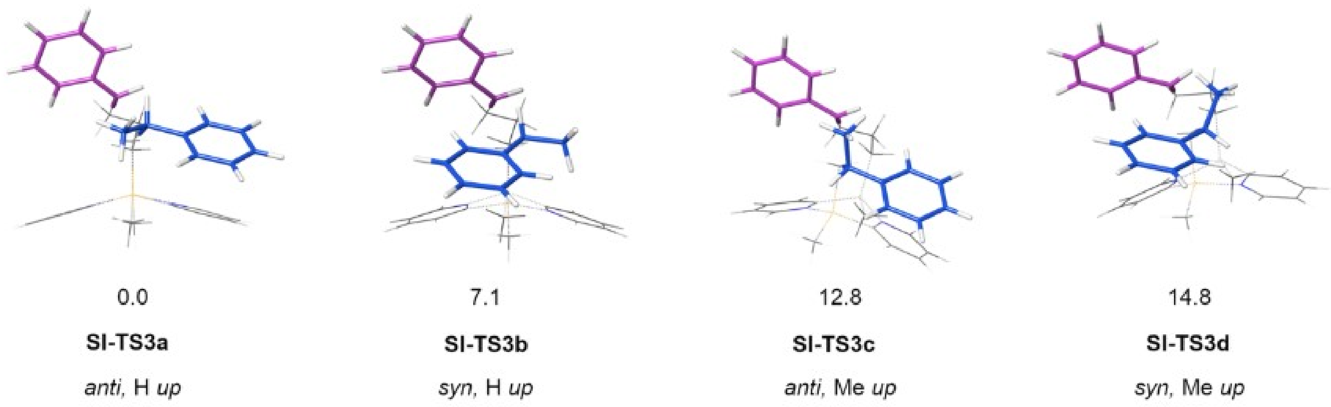

Figure S31. Isomeric transition states illustrating effect of conformation on **TS3** energy, energies are relative electronic energies in kJ mol^{-1}.

Energies calculated at B3LYP-6-31G(d) level

Structures **SI-TS3c** and **SI-TS3d** show a higher energy TS with a bulkier group projecting towards the dimer chain.

Enthalpy profile for polymerisation

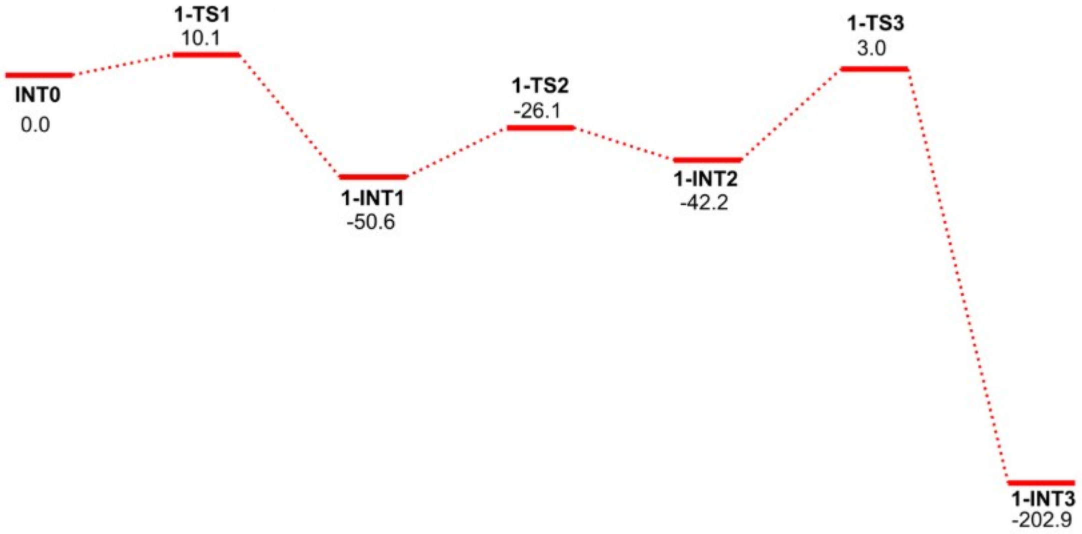

Figure S32. Enthalpy profile for polymerisation reaction. Energies in kJ mol^{-1}.

NBO calculations

NBO (v3.1)[18] was used to analyse the nature of **1-INT2** following SPE calculation using ωB97X-D/6-311++G(d,p). Second order analysis revealed strong interactions between the C=C π bond of styrene and AOs on Al suggesting the structure is more accurately described as a π-complex than a species with an Al-C σ bond.

a)

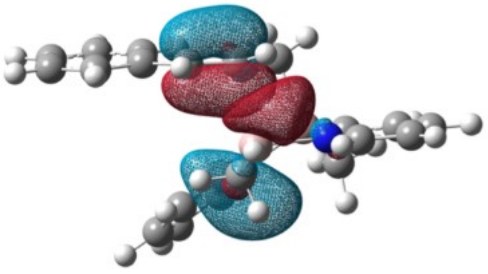

b)

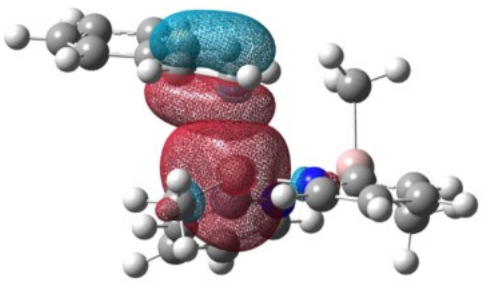

Figure S33. Overlap of the C=C π NBO with a) Al p NAO, b) Al s NAO

Relevant NBO output:

Donor NBO	Acceptor NBO	E(2) kcal/mol
38. BD (2) C 18 – C 19	96. LP*(1)Al 8	42.34
38. BD (2) C 18 - C 19	98. LP*(3)Al 8	29.24

Scans for barrierless steps

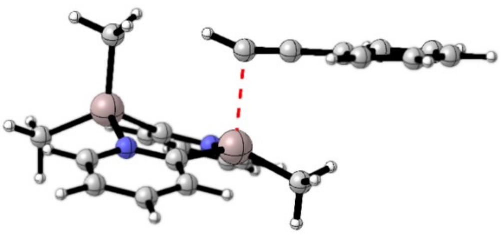

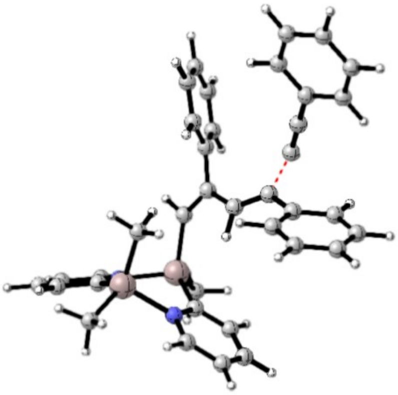

Bond scan for **INT1** to **2-INT2**

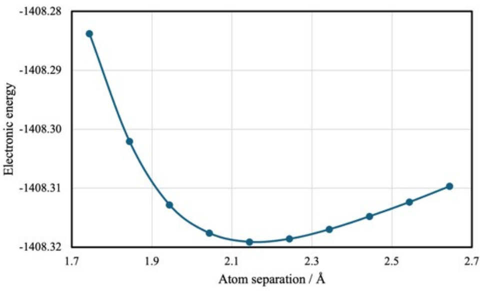

Bond scan for **2-INT3** to **2-INT4**

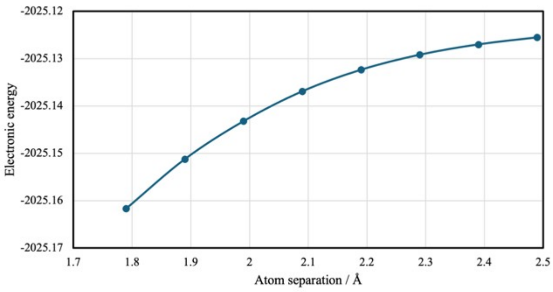

Ring closing

A conformational search of **2-INT4** was carried out using MacroModel and distances between atoms 18, 69 and 18, 70 were calculated. The low energy conformer with distances less than 4 Å was selected for DFT optimisation. *Conformer 3 required a ring flip of the catalyst structure centre and was ignored.

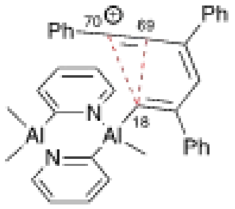

Conformer #	Relative Potential Energy-S-OPLS/ kJ mol^{-1}	Distance 18-69	Distance 18-70
1	0	4.082	5.421
2	0.608	4.083	5.424
3*	1.701	3.508	4.814
**4**	**1.771**	**3.575**	**4.876**
5	1.820	3.602	4.914
6	1.923	3.445	4.745
7	2.002	3.715	5.033
8	2.477	3.791	5.114
9	3.202	3.952	5.192
10	3.348	3.929	5.117
11	3.932	3.670	4.846
12	4.224	3.367	4.520

13	4.515	3.550	4.857
14	4.546	3.668	4.817
15	5.003	3.544	4.853
16	5.229	4.038	5.376
17	5.676	3.488	3.735
18	5.835	3.934	5.272
19	6.012	3.791	5.119
20	6.079	4.004	5.344
21	6.281	4.017	3.822
22	6.762	3.872	5.204
23	6.834	4.013	5.352
24	7.665	4.028	3.811
25	9.078	3.512	4.830
26	9.810	3.483	4.692
27	10.910	3.574	3.966
28	11.061	3.479	4.588
29	11.166	3.420	3.510
30	11.340	3.455	4.662
31	12.328	3.444	3.672
32	12.651	3.542	3.920
33	12.862	4.042	3.835
34	13.255	3.997	3.757
35	13.817	3.585	3.792
36	14.295	3.412	4.527

37	14.516	3.575	4.716
38	15.169	4.252	4.139
39	15.577	4.244	4.155
40	16.374	3.493	3.550
41	17.050	4.220	4.162
42	17.095	4.166	5.461
43	17.440	4.245	4.186
44	18.456	3.464	4.754
45	19.200	3.888	5.218
46	19.219	4.130	5.467
47	19.541	3.757	4.489
48	19.552	3.519	4.788
49	20.312	3.827	5.154
50	20.628	3.457	4.641

Alternative regioselectivity

Carbene insertion leading to 1,2,4 product (2-TS3)	Carbene insertion leading to 1,3,5 product

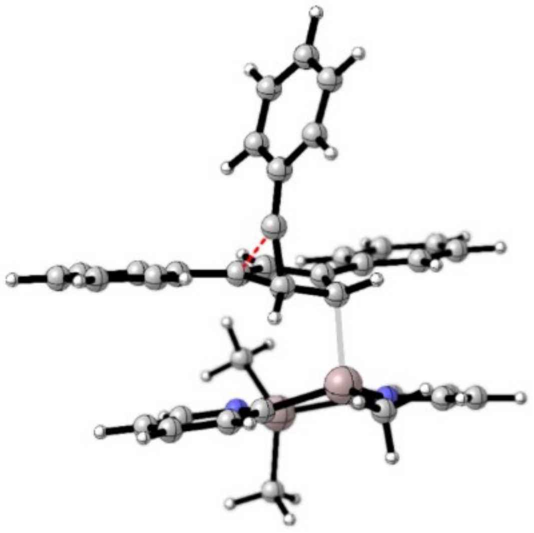

E = –2025.263689

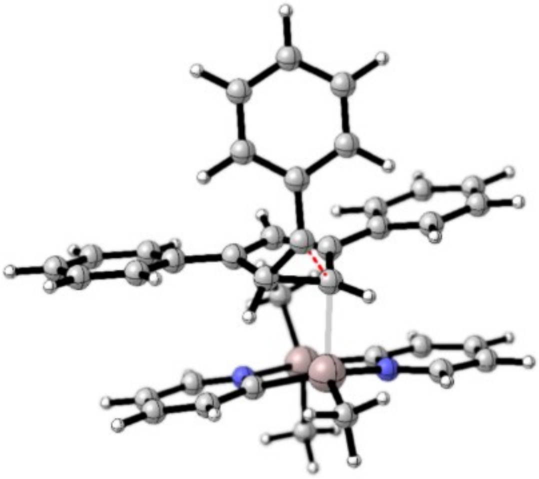

E = –2025.262613

Energies calculated at B3LYP-D3-6-31G(d) level

Geometry of alkene

Geometry optimisation and single point energy calculated at B3LYP/6-31G(d) level.

***trans* isomer**	***cis* isomer**
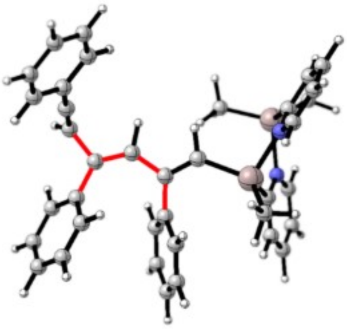	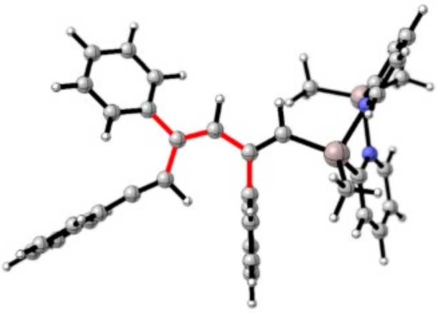
E = –2025.179077	E = –2025.189088

Trimerisation pathway reproduced

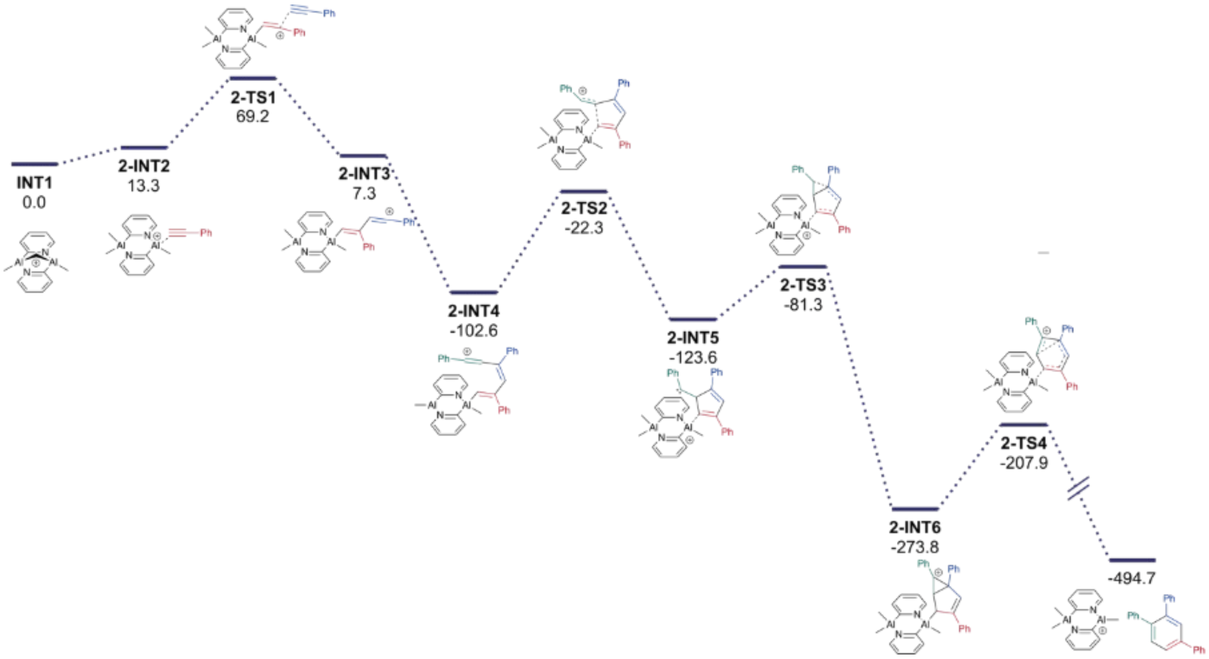

Elimination pathway

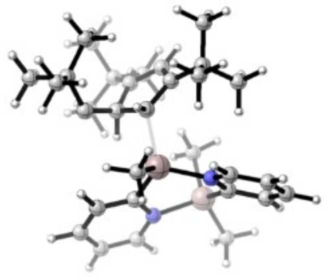

t_butyl_carbene

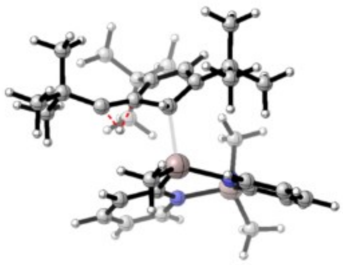

t_butyl_h_shift_ts

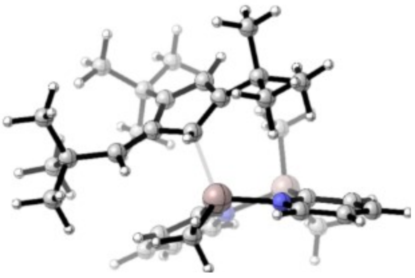

t_butyl_fulvene

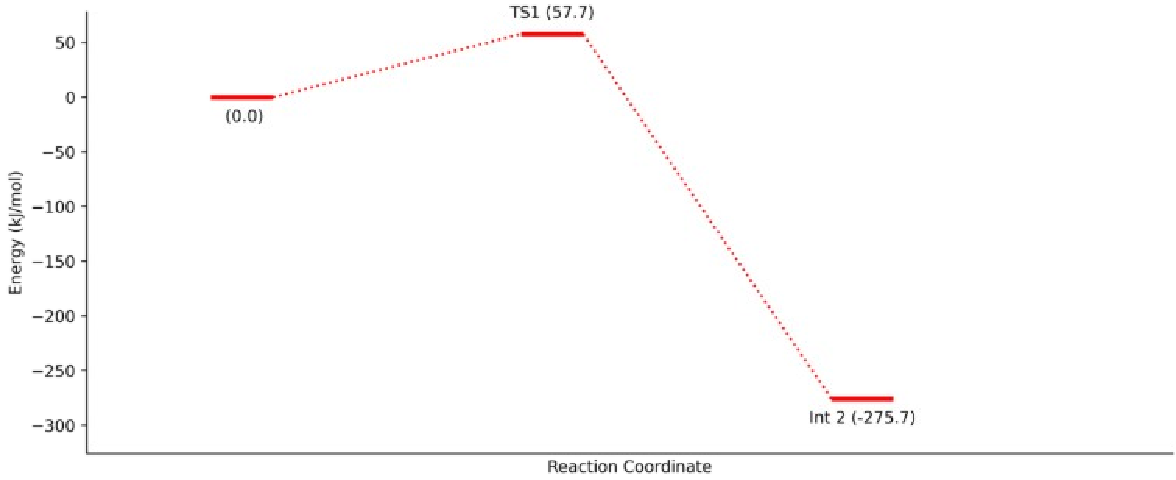

Figure S34. Free energy profile for 1,2-H shift step in formation of fulvene product in t-butylacetylene trimerisation.

Structure of Apollo repository

Output files for DFT calculations are deposited in an Apollo repository[19] at https://doi.org/10.17863/CAM.121268.

```
├── Acetylene_trimerisation
│   ├── 01_INT1.out
│   ├── 02_2-INT2.out
│   ├── 03_2-TS1.out
│   ├── 04_2-INT3.out
│   ├── 05_2-INT4.out
│   ├── 06_2-TS2.out
│   ├── 07_2-INT5.out
│   ├── 08_2-TS3.out
│   ├── 09_2-INT6.out
│   ├── 10_2-TS4.out
│   └── 124_triphenylbenzene_reference.out
├── SI
│   ├── 135_carbene_insertion.out
│   ├── Elimination_pathway
│   │   ├── t_butyl_carbene.out
│   │   ├── t_butyl_fulvene.out
│   │   └── t_butyl_h_shift_ts.out
│   ├── FIA
│   │   ├── AlCl3_F.out
│   │   ├── AlCl3.out
│   │   ├── CF2O_F.out
│   │   ├── CF2O.out
│   │   ├── INT1_F.out
│   │   ├── INT1.out
│   │   ├── trityl_F.out
│   │   └── trityl.out
│   ├── Geometry_alkene
```

```
│   │   ├── E_geometry_trimer.out
│   │   └── Z_geometry_trimer.out
│   ├── INT1_nbo.out
│   ├── Scans_for_barrierless_steps
│   │   ├── scan_2-INT3_to_2-INT4.out
│   │   └── scan_INT1_to_2-INT2.out
│   └── Stereochemistry_chain_transfer
│       ├── SI-TS3a.out
│       ├── SI-TS3b.out
│       ├── SI-TS3c.out
│       └── SI-TS3d.out
├── Stereochemistry_TS3
│   ├── 1-TS3a.out
│   ├── 1-TS3a_prime.out
│   ├── 1-TS3b.out
│   ├── 1-TS3b_prime.out
│   ├── 1-TS3_geom.out
│   └── 1-TS3_prime_geom.out
└── Styrene_polymerisation
    ├── 1_1-INT1.out
    ├── 2_1-TS1.out
    ├── 3_1-INT2.out
    ├── 4_1-TS2.out
    ├── 5_1-INT2.out
    ├── 6_1-TS3.out
    └── 7_1-INT3.out
```

References

1. C. Xi, Z. Sun and Y. Liu, *Dalton Trans.*, 2013, **42**, 13327–13330
2. M. J. Frisch, G. W. Trucks, H. B. Schlegel, G. E. Scuseria, M. A. Robb, J. R. Cheeseman, G. Scalmani, V. Barone, G. A. Petersson, H. Nakatsuji, X. Li, M. Caricato, A. V. Marenich, J. Bloino, B. G. Janesko, R. Gomperts, B. Mennucci, H. P. Hratchian, J. V. Ortiz, A. F. Izmaylov, J. L. Sonnenberg, D. Williams-Young, F. Ding, F. Lipparini, F. Egidi, J. Goings, B. Peng, A. Petrone, T. Henderson, D. Ranasinghe, V. G. Zakrzewski, J. Gao, N. Rega, G. Zheng, W. Liang, M. Hada, M. Ehara, K. Toyota, R. Fukuda, J. Hasegawa, M. Ishida, T. Nakajima, Y. Honda, O. Kitao, H. Nakai, T. Vreven, K. Throssell, J. A. Montgomery, Jr., J. E. Peralta, F. Ogliaro, M. J. Bearpark, J. J. Heyd, E. N. Brothers, K. N. Kudin, V. N. Staroverov, T. A. Keith, R. Kobayashi, J. Normand, K. Raghavachari, A. P. Rendell, J. C. Burant, S. S. Iyengar, J. Tomasi, M. Cossi, J. M. Millam, M. Klene, C. Adamo, R. Cammi, J. W. Ochterski, R. L. Martin, K. Morokuma, O. Farkas, J. B. Foresman, and D. J. Fox, Gaussian 16, Revision B.01, Gaussian, Inc., Wallingford CT, 2016.
3. A. D. Becke, *Phys. Rev. A*, 1988, **38**, 3098–310.
4. C. Lee, W. Yang and R. G. Parr, *Phys. Rev. B*, 1988, **37**, 785–789.
5. A. D. Becke, *J. Chem. Phys.*, 1993, **98**, 5648–5652.
6. S. Grimme, S. Ehrlich and L. Goerigk, *J. Comput. Chem.*, 2011, **32**, 1456–1465.
7. J.-D. Chai and M. Head-Gordon, *Phys. Chem. Chem. Phys.*, 2008, **10**, 6615–6620.
8. Schrödinger Release 2024-3: Maestro, Schrödinger, LLC, New York, NY, 2021.
9. CYLview, 20; Legault, C. Y., Université de Sherbrooke, 2020 (http://www.cylview.org).
10. (a) F. Neese, *WIRES Comput. Molec. Sci.*, 2025, **15**, e70019; (b) F. Neese, *J. Comput. Chem.*, 2023, **44**, 381–396
11. Y.-S. Lin, G.-D. Li, S.-P. Mao and J.-D. Chai, *J. Chem. Theory Comput*., 2013, **9**, 263–272.
12. F. Weigend and R. Ahlrichs, *Phys. Chem. Chem. Phys*., 2005, **7**, 3297–3305.
13. S. Grimme, J. Antony, S. Ehrlich and H. Krieg, *J. Chem. Phys.*, 2010, **132**, 154104
14. (a) T. Lu and F. Chen, *J. Comput. Chem.*, 2012, **33**, 580–592; (b) T. Lu, *J. Chem. Phys.*, 2024, **161**, 082503.
15. T. Lu and F. Chen, *J. Mol. Graph. Model.*, 2012, **38**, 314–323.
16. E. C. Meng, T. D. Goddard, E. F. Pettersen, G. S. Couch, Z. J. Pearson, J. H. Morris and T. E. Ferrin, *Protein Sci.*, 2023, **32**, e4792.
17. (a) L. Falivene, R. Credendino, A. Poater, A. Petta, L. Serra, R. Oliva, V. Scarano and L. Cavallo, *Organometallics*, 2016, **35**, 2286–2293; (b) L. Falivene, Z. Cao, A. Petta, L. Serra, A. Poater, R. Oliva, V. Scarano and L. Cavallo, *Nat. Chem.*, 2019, **11**, 872–879.
18. NBO Version 3.1, E. D. Glendening, A. E. Reed, J. E. Carpenter, and F. Weinhold.
19. Choudhury, D., Danylyuk, R., Terzopoulos, A., Potter, N., Scherman, O., Goodman, J., & Wright, D. (2025). Research data supporting "Oligomerisation and Stereoselective Polymerisation of Alkenes and Alkynes Using Pyridyl-based Al(III) Catalysts". Apollo - University of Cambridge Repository. https://doi.org/10.17863/CAM.121268.
